# Local Victory: Assessing Interspecific Competition in Seagrass From a Trait-Based Perspective

**DOI:** 10.3389/fpls.2021.709257

**Published:** 2021-11-02

**Authors:** Agustín Moreira-Saporiti, Sonia Bejarano, Inés G. Viana, Elizabeth Fay Belshe, Matern S. P. Mtolera, Mirta Teichberg

**Affiliations:** ^1^Algae and Seagrass Ecology Group, Department of Ecology, Leibniz Centre for Tropical Marine Research, Bremen, Germany; ^2^Faculty for Biology and Chemistry, University of Bremen, Bremen, Germany; ^3^Reef Systems Research Group, Department of Ecology, Leibniz Centre for Tropical Marine Research, Bremen, Germany; ^4^Centro Oceanográfico de A Coruña (IEO, CSIC), A Coruña, Spain; ^5^Institute of Marine Sciences, University of Dar es Salaam, Zanzibar Archipelago, Tanzania

**Keywords:** resource preemption, space preemption, trait-based approach, traits ordination, tropical seagrass

## Abstract

Tropical seagrass meadows are formed by an array of seagrass species that share the same space. Species sharing the same plot are competing for resources, namely light and inorganic nutrients, which results in the capacity of some species to preempt space from others. However, the drivers behind seagrass species competition are not completely understood. In this work, we studied the competitive interactions among tropical seagrass species of Unguja Island (Zanzibar, Tanzania) using a trait-based approach. We quantified the abundance of eight seagrass species under different trophic states, and selected nine traits related to light and inorganic nutrient preemption to characterize the functional strategy of the species (leaf maximum length and width, leaves per shoot, leaf mass area, vertical rhizome length, shoots per meter of ramet, rhizome diameter, roots per meter of ramet, and root maximum length). From the seagrass abundance we calculated the probability of space preemption between pairs of seagrass species and for each individual seagrass species under the different trophic states. Species had different probabilities of space preemption, with the climax species *Thalassodendron ciliatum, Enhalus acoroides, Thalassia hemprichii*, and the opportunistic *Cymodocea serrulata* having the highest probability of preemption, while the pioneer and opportunistic species *Halophila ovalis, Syringodium isoetifolium, Halodule uninervis*, and *Cymodocea rotundata* had the lowest. Traits determining the functional strategy showed that there was a size gradient across species. For two co-occurring seagrass species, probability of preemption was the highest for the larger species, it increased as the size difference between species increased and was unaffected by the trophic state. Competitive interactions among seagrass species were asymmetrical, i.e., negative effects were not reciprocal, and the driver behind space preemption was determined by plant size. Seagrass space preemption is a consequence of resource competition, and the probability of a species to exert preemption can be calculated using a trait-based approach.

## Introduction

Seagrasses are a polyphyletic assemblage of angiosperm plants that inhabit coastal areas and undergo their entire life cycle in the water. Their ecosystems are highly productive habitats that support considerable biomasses of associated species diversity (Orth et al., 1984) and offer highly valued ecosystem services (Costanza and Folke, 1997), including climate regulation (Duarte and Chiscano, 1999) and nutrient filtering (Hemminga et al., 1991). These habitats are confined to a thin strip of shallow nearshore waters (Olsen et al., 2016) that presently are susceptible to increasingly high anthropogenic pressures, both at a local and global scale. Leading anthropogenic factors include eutrophication that reduces light by the increase in phytoplankton and opportunistic macroalgae biomass (Cardoso et al., 2004; Schmidt et al., 2012). Global warming directly affects seagrasses through thermal stress (Ontoria et al., 2019), reducing their biomass and eventually causing mortality (Rasheed and Unsworth, 2011). The influence of these drivers results in a decline in the coverage of seagrass worldwide (Orth et al., 2006; Waycott et al., 2009; Unsworth et al., 2018). Although this trend may be reversed in temperate and sub-tropical areas (de los Santos et al., 2019; Schäfer et al., 2021), the situation in tropical areas is largely underreported.

The seagrass communities present in a specific habitat are the product of several filters that generally act in a hierarchical fashion (Garnier et al., 2016). Firstly, seagrass dispersion methods control the potential of a species to colonize a new habitat (Orth et al., 2007; McMahon et al., 2014). Secondly, abiotic filters determine which seagrass species can establish given the local environmental conditions, availability of resources, and disturbance regime (Wilson, 2011). Lastly, the subset of species that have successfully colonized an area will interact and compete with each other for resources (Tilman, 1985). All these processes filter the regional species pool and ultimately assemble local seagrass communities (Keddy, 1992).

Seagrasses, similarly to terrestrial plant communities, undergo successional states from pioneer to climax species (Young and Kirkman, 1975; Birch and Birch, 1984; Williams, 1987, 1990; Fourqurean et al., 1995; Davis and Fourqurean, 2001). Pioneer species within seagrass meadows are generally small and fast growing, whereas climax species are large and slow-growing. If conditions are not extrinsically disturbed, succession should follow a direction, which has been long believed to end in a monospecific meadow formed by a climax seagrass species (Moliner and Picard, 1952; Aleem, 1955; den Hartog, 1971, 1977; Zieman, 1982). Competitive interactions between pioneer and climax seagrass species are the mechanisms driving the course of the succession (Connell and Slatyer, 1977; Tilman, 1994). In plants, interspecific competition can be broadly defined as the limiting effect that species may have on each other, directly or indirectly reducing or preventing growth and survival (Connell, 1990; Vilà and Sardans, 1999).

Since the first descriptions of seagrass meadows, there were reports of potential interspecific competition between species. Competition for space between *Posidonia oceanica* and *Cymodocea nodosa* was early reported in the Mediterranean Sea (Moliner and Picard, 1952; Aleem, 1955), as well as the dominance of *Thalassia testudinum* over other seagrasses present in the coasts of Florida (USA, Phillips, 1960), the competition between *Ruppia* sp. and other seagrasses (den Hartog, 1970), or the dominance of *Thalassia hemprichii* in the intertidal area over *Halodule uninervis* (Lan et al., 2005). *Zostera marina* suppresses shoot production of *Z. japonica* (Nomme and Harrison, 1991a,b) and *Halophila stipulacea* can displace *Syringodium filiforme* from its native habitat (Willette and Ambrose, 2012; Viana et al., 2019a). The observation of competition prompted the classification of seagrass species according to their life-history strategies. For instance, Harrison (1979) classified *Z. marina* as a k-strategist and *Z. japonica* as an r-strategist due to their differential investment on maintenance of belowground and reproductive structures, respectively. Birch and Birch (1984) provided a detailed description of succession in a seagrass meadow after a hurricane, providing insight into how the successional states are dominated by seagrass with differential life-history strategies, suggesting that succession is directional and not probabilistic.

Competition happens when there are resources that are preemptable and limited (Tilman, 1985). Preemptable resources are those that, once taken by an organism, are not available for the others (Underwood and Denley, 1984). Light is, therefore, a preemptable resource for plants (Schwinning and Weiner, 1998). Particularly, in the case of seagrasses, shading has been proposed as one of the main underlying mechanisms for space competition (Turner, 1983, 1985) also common in giant kelp forests (Rosenthal et al., 1974). The canopy of *T. testudinum* blocks up to 75% of the light reaching it (Zieman et al., 1984), and *Z. marina* can competitively exclude *Ruppia maritima* by light shading (Orth, 1977), among other examples (Fourqurean et al., 1995; Duarte et al., 1998). Shading is one of the mechanisms through which *T. testudinum* displaces *S. filiforme*, with the larger leaves of *T. testudinum* commonly intercepting light that otherwise would reach *S. filiforme* (Williams, 1987). These reports indicate that the preemption of light is heavily influenced by morphological aboveground traits related to plant size.

Inorganic nutrients are also a preemptable resource for seagrasses (Williams, 1987). Seagrasses obtain inorganic nutrients from both the pore water in the sediments and the water column (Iizumi and Hattori, 1982; Thursby and Harlin, 1982; Short and McRoy, 1984; Williams and Ruckelshaus, 1993; Viana et al., 2019b) and can often be nutrient-limited in tropical areas (Orth, 1977; Bulthuis and Woelkerling, 1981; Short et al., 1985; Powell et al., 1989; Duarte et al., 1995; Agawin et al., 1996). Therefore, the characteristics of aboveground and rhizomatic structures can influence seagrass competition for nutrients in the water column and in the sediments, respectively. In competition between *Ruppia maritima* and *Halodule wrightii*, for example, involvement of belowground nutrients has been shown (Pulich Jr, 1985). Additionally, other studies suggest that nutrient competition among seagrasses can occur and is affected by the characteristics of their rhizomes and roots (Fourqurean et al., 1995; Duarte et al., 2000; Bando, 2006).

Interspecific seagrass competition for light and inorganic nutrients appears to be asymmetric, whereby a species affects another but with no reciprocity in the effect (Connell, 1983; Schoener, 1983; Schwinning and Weiner, 1998; Davis and Fourqurean, 2001). Duarte et al. (2000) suggest that differences in plant size among species may be partly responsible for this phenomenon (see also Vermaat et al., 1995; Terrados et al., 1999). More recent studies show that competition for light and nutrients happen simultaneously, limiting the possibility to separate their effects in the field (Fourqurean et al., 1995; Duarte et al., 1997, 2000; Nakaoka and Iizumi, 2000; Davis and Fourqurean, 2001; Taplin et al., 2005). Additionally, species may exploit resources differently. Seagrasses show considerable vertical stratification within the sediment with a tendency of larger species to extend deeper into the sediments than smaller ones (Duarte et al., 1998). Therefore, although these plants co-occur in aboveground space, they do not share the same belowground space (Williams, 1990; Duarte et al., 2000; Ooi et al., 2011), suggesting the possibility of belowground niche differentiation among seagrass species (Meilhac et al., 2020). Competition for resources should therefore be fundamentally determined by the traits of coexisting plants (Hofman and Ennik, 1980; Firbank and Watkinson, 1987; Schwinning and Weiner, 1998), yet this remains to be tested in seagrass communities.

Mixed seagrass meadows are a common feature in tropical seascapes, challenging the general hypothesis that final successional stages could be monopolized by a single species out-competing the rest (Young and Kirkman, 1975). Unlike other terrestrial and marine assemblages, colonizing and middle-successional seagrass species are not confined to patch mosaics within mature seagrass assemblages, but occur as individuals scattered throughout, posing the question as to how a multispecies meadow can be maintained (Williams, 1990). The environmental conditions in which seagrass meadows develop can, however, favor specific species when it comes to interspecific competition. In the field, *Z. japonica* dominates the intertidal area, much more prone to disturbances, whereas *Z. marina* is more abundant in the subtidal zone (Harrison, 1979). Nutrient enrichment changes the dominance of seagrass communities from *T. testudinum* to *H. wrightii* (Fourqurean et al., 1995). Seaweed farming can, through trampling, favor the dominance of seagrass over benthic macroalgae (Moreira-Saporiti et al., 2021). Seagrass plants respond to varying environmental conditions and levels of stress through their traits (Roca et al., 2016). This is the case for tropical seagrass species in controlled experiments, in which temperature and nutrient enrichment affected and changed their morphological, biochemical, and photo-physiological traits (Mvungi and Pillay, 2019; Artika et al., 2020), with a majority of these responses being species-specific (Viana et al., 2020). It is therefore expected that a change in the traits of seagrass species under different levels of anthropogenic pressure (e.g., eutrophication) may affect the outcome of interspecific competition.

Traits are defined as “any morphological, physiological, or phenological heritable feature measurable at the individual level, from the cell to the whole organism, without reference to the environment or any other level of organization” (Violle et al., 2007 as modified by Garnier et al., 2016). The study of traits, therefore, allows us to understand the relationships between organisms from a functional perspective. Trait-based ecology in fact assumes that structures at higher organizational scales are largely a result of the composite traits of the individuals (Grime, 1998; Shipley et al., 2016). One of the main tools used in functional trait-based studies is the construction of a multidimensional space where axes are ecologically relevant traits or linear combinations of a set of traits of a species or a community of species (Mouillot et al., 2013). The coordinates of the species in the multidimensional space are, therefore, determined by its traits. Although trait-based approaches can be applied to all kinds of organisms, these are currently most developed for terrestrial plants (Lavorel and Garnier, 2002; Garnier et al., 2016), but are also used for marine organisms (Litchman and Klausmeier, 2008; Andersen and Pedersen, 2009; Litchman et al., 2010, 2013; Elleouet et al., 2014). Trait-based approaches have been rarely, but successfully, applied in seagrass communities, with results suggesting that functional traits underpin community-level primary production (Jänes et al., 2017; Gustafsson and Norkko, 2019) or mediate herbivory and predation in seagrass ecosystems (Pagès et al., 2012; Lewis and Boyer, 2014).

This study addresses our incipient understanding of competition among tropical seagrass species (Ooi et al., 2011) and the role traits play in competitive outcomes. Specifically, we aim to quantify the extent to which seagrass traits (known to correlate with their ability to compete for light and nutrients) affect the probability of space preemption by seagrass species under different trophic states. For this purpose, we (i) quantified the abundance of the seagrass species off Unguja Island (Zanzibar Archipelago, Tanzania) in sites subject to varying trophic states and examined pairwise space preemption of seagrass at the local level, (ii) ordered the species according to nine traits informative of their functional strategy during interspecific competition for inorganic nutrients and light, (iii) examined the effect of the trophic states on seagrass traits, and (iv) tested the relationship of the difference between the functional strategies of pairs of seagrass species and their probability of space preemption. We hypothesized that a species' functional strategy will have an effect in its preemptive ability, and this effect may change under different trophic states.

## Materials and Methods

### Study Area

**Figure 1:**
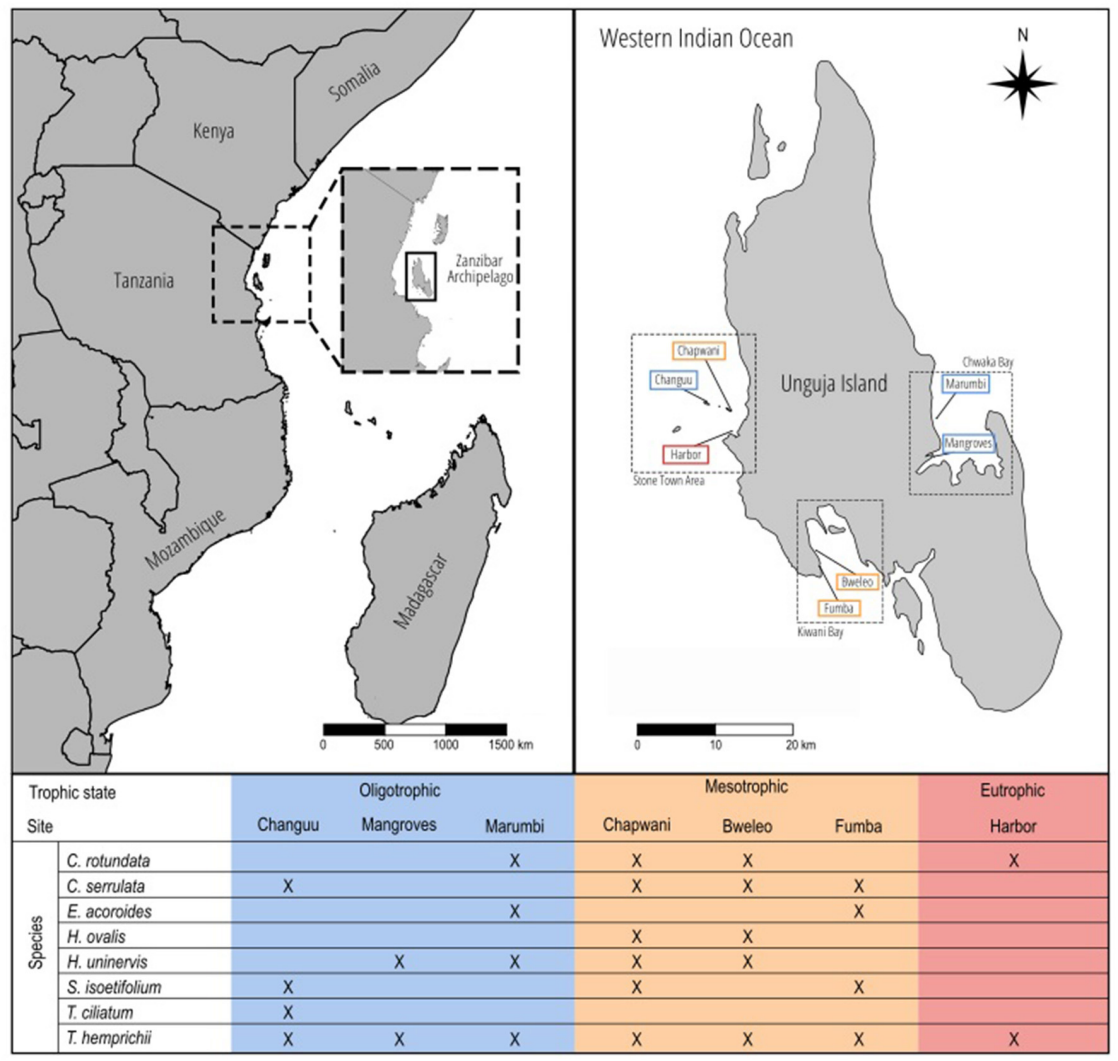
Map of the study area. On the top left, an overview of the Eastern African coast, where the Zanzibar Archipelago is located. On the top right, Unguja Island and the seven sites selected in three different areas around the island. The colors indicate the trophic state to which they were assigned. The bottom table indicates which seagrass species were present in which sites.

Unguja Island is the most populated island in the tropical archipelago of Zanzibar (~900.000 inhabitants), off the coast of Tanzania in the Western Indian Ocean (Figure 1), and one of the main hotspots of seagrass biodiversity in the world (Short et al., 2007). Seagrass communities are mainly formed by eight species: *Cymodocea rotundata, Cymodocea serrulata, Enhalus acoroides, Halophila ovalis, Halodule uninervis, Syringodium isoetifolium, Thalassodendron ciliatum*, and *Thalassia hemprichii*. We surveyed seven different sites (Figure 1) expected to show different trophic states. Each of the seven study sites comprised a subtidal seagrass meadow of ~10,000 m^2^ generally bounded by a coastal rocky or sandy area and a fringing coral reef. All seven study sites were surveyed in November 2016.

### Characterization of the Trophic States

We surveyed different environmental variables in order to determine the trophic states of the sampling sites (Burkholder et al., 2007). The indicators selected were: macroalgae biomass (g DW m^−2^), chlorophyll-*a* in the water column (μg l^−1^), sediment δ^15^N (‰) and concentrations of dissolved inorganic nitrogen (DIN), and ${\text{PO}}_{4}^{3-}$ in pore water (μM).

#### Macroalgae Biomass

Macroalgae biomass was quantified along five 50-m transects per site, set perpendicular to the coast and parallel to each other, separated by ~50 m. We collected the macroalgae present in three random 0.25 × 0.25 m quadrats per transect. The macroalgae samples were cleaned of sediments and rinsed with water. They were then dried at 50°C in a forced air oven until constant dry weight (g DW). The macroalgae biomass was calculated as the g DW divided by the area of the quadrat (g DW m^−2^).

#### Chlorophyll-a in the Water Column

In the proximities of each transect, we collected five ~3-L seawater samples from each site and kept them in a cooler box until filtration. Seawater was immediately filtered upon arrival in the Institute of Marine Sciences (IMS, Stone Town, Zanzibar) under constant pressure onto pre-combusted (5 h, 450°C) and pre-weighed Whatman GF/F filters (0.45-μm pore size). The filters were stored at −20°C and transported frozen to the Leibniz Centre for Tropical Marine Research in Bremen (ZMT) (Germany). Chlorophyll-*a* was extracted from the filters in 8 ml of 96% ethanol in glass vials heated for 5 min at 80°C, covered with aluminum foil, and placed in a rotor at room temperature for ~24 h. Extracts were subsequently centrifuged at 5,000 rpm for 20 min. Chlorophyll-*a* samples were determined in a Shimadzu UV-1700 photometer, and calculated as μg l^−1^.

#### DIN and ${\text{PO}}_{4}^{3-}$ Concentrations in Pore Water

We took one sediment pore water sample per transect from each site using 30-cm PVC cores. The cores were pushed into the sediments, and after extraction, a rhizon soil moisture sampler (Eijkelkamp Soil & Water, Netherlands) connected to a 20-ml syringe was placed in a hole corresponding to a depth of 5 cm below the sediment surface. Making a vacuum with the syringe, the water was pulled out of the sediment cores. These samples were immediately filtered (0.45-μm pore size, Whatman GF/F filters) in pre-rinsed polyethylene bottles, frozen (−20°C), and transported to the ZMT. Analysis was performed using a continuous flow injection analyzing system (Skalar SAN++-System) following Grasshoff et al. (1983). The measuring procedure had a relative standard deviation < 3.5% with reference to the linear regression of an equidistant 10-point calibration line from NIST standards.

#### δ^15^N in the Sediment

We took one 50-ml surface sediment sample per transect for δ^15^N analysis. The samples were stored at −20°C and transported frozen to the ZMT. They were then dried at 50°C in a forced air oven until constant DW, ground to a fine powder with mortar and pestle, and weighed into tin capsules prior to analysis for nitrogen stable isotope composition (δ^15^N) with a gas isotope ratio mass spectrometer (Thermo Finnigan Delta Plus, Waltham, MA, USA). Results are expressed in δ notation (‰) where the standard for δ^15^N is atmospheric N_2_.

### Characterization of the Seagrass Species and Their Traits

#### Measurement of Seagrass Cover and Pairwise Space Preemption per Species

**Figure 2:**
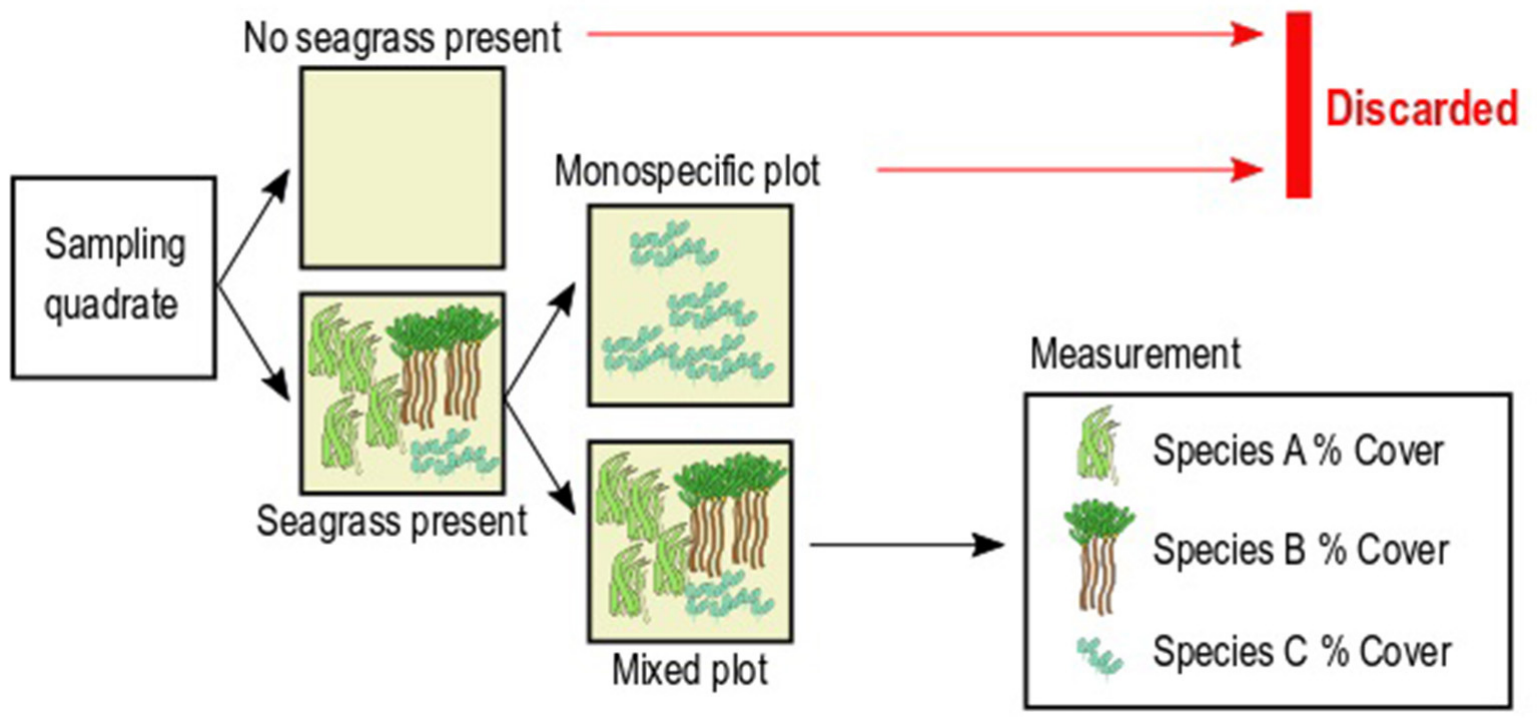
Schematic representation of the sampling design to measure seagrass cover percentage.

Seagrass cover was quantified along five 50-m transects per site, set perpendicular to the coast and parallel to each other, separated by ~50 m. Seagrass cover was visually quantified as the area percentage occupied per species within seagrass plots of 0.5 × 0.5 m marked by PVC quadrats, randomly placed along each transect (*n* = 9 per transect, 45 per site). The total cover, including bare sediment, was bound to 100%. In order to assess the abundance of seagrass and the competitive outcome between species, we selected only the plots in which at least two seagrass species were present (Figure 2). We discarded any plot in which seagrass species were absent to eliminate the confounding effect of presence/absence of seagrass, which is also governed by factors other than competition (e.g., colonization success) (see Tilman, 1985; Keddy, 1992; Garnier et al., 2016). We also discarded any plot in which only one species was present in order to measure abundance only in plots where competitive interactions were occurring (Figure 2). The goal of this step is to ensure that the seagrass abundance in the resulting plots can only be attributed to the outcome of interspecific interactions. The resulting number of plots differed between species: *C. rotundata* (*n* = 35), *C. serrulata* (*n* = 58), *E. acoroides* (*n* = 18), *H. ovalis* (*n* = 24), *H. uninervis* (*n* = 64), *S. isoetifolium* (*n* = 45), *T. ciliatum* (*n* = 19), and *T. hemprichii* (*n* = 127).

Per plot and species, we calculated the cover percentage and pairwise space preemption. We calculated the pairwise space preemption following Equation 1:


(1) $$\begin{array}{l} \text{Space }{\text{preemption}}_{\text{SpeciesA}}\text{= }\%{\text{Cover}}_{\text{SpA}}\text{- }\%{\text{Cover}}_{\text{SpB}}\;\\ \text{ =}\{\begin{array}{c} 1\;if\;\%{\text{Cover}}_{\text{SpA}}>\%{\text{Cover}}_{\text{SpB}}\\ 0\;if\;\%{\text{Cover}}_{\text{SpA}}\leq \%{\text{Cover}}_{\text{SpB}} \end{array} \end{array}$$

Pairwise space preemption was computed here as the difference between the cover percentage of a given seagrass species (A) minus the cover percentage of a second species (B) sharing the plot (Figure 2). Pairwise space preemption was then converted into a binary variable taking a value of “1” where the cover percentage of Species A was higher than cover percentage of Species B (preemption by A), and “0” where the cover percentage of Species A was equal or lower than the cover percentage of Species B (no preemption by A). This calculation was repeated with each species sharing the plot with Species A. Lastly, the probability of preemption by Species A was calculated as the number of successes (preemption) divided by the total number of plots in which Species A was present (Equation 2).


(2) $$\begin{array}{l} \text{Probability of space }{\text{preemption}}_{\text{SpeciesA}}\;\\ \text{ =}\frac{\text{Number of }{\text{successes}}_{\text{Species A}}}{\text{Total number of }{\text{plots}}_{\text{Species A}}} \end{array}$$

#### Sampling and Measurement of Traits Linked to Resource Competition

**Figure 3:**
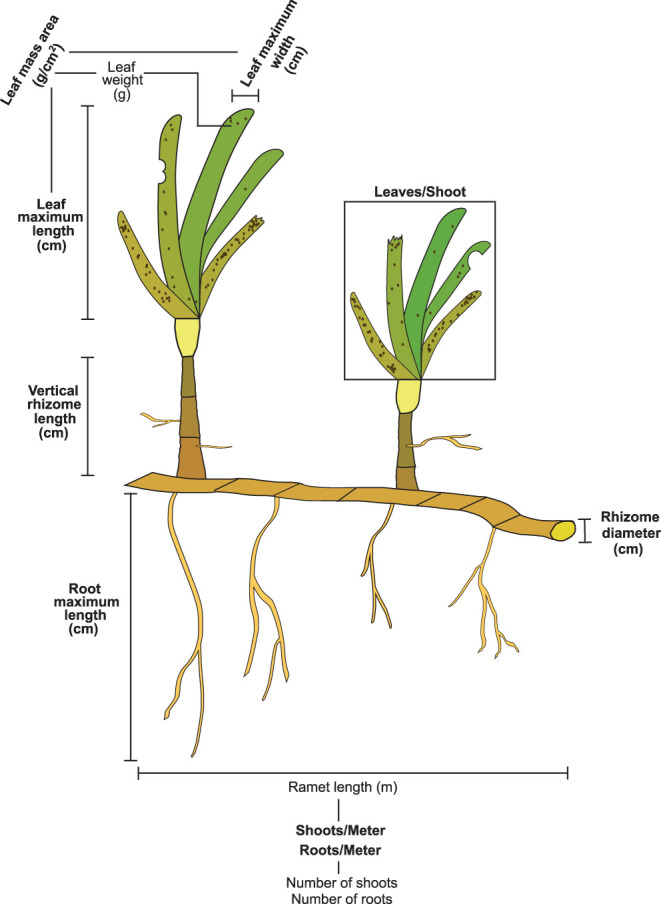
Generic representation of a seagrass ramet and the traits measured. Traits included in the analysis are highlighted in bold. Traits that are not in bold were necessary in order to calculate the functional traits included in the analysis.

Finding explicit links between traits and interspecific competition for resources proved a difficult task due to the lack of studies addressing this question. We considered nine traits reportedly correlated to light and inorganic nutrients preemption (Figure 3 and Table 1), namely, leaf maximum length (leaf ML, cm), leaf maximum width (leaf MW, cm), vertical rhizome length (VR length, cm), leaves per shoot (leaves/Sh, leaves shoot^−1^), rhizome diameter (RhD, cm), roots per meter of seagrass ramet (roots/M, roots meter^−1^), root maximum length (Root ML, cm), leaf mass area (LMA, g cm^−2^), and shoots per meter of seagrass ramet (shoots/M, shoots m^−1^). For further clarification, traits were classified into two groups: canopy forming and belowground structure traits. The references cited in Table 1 indicate competition for resources, and they propose traits that could be responsible for the competition for said resource. We did not find references addressing competition for inorganic nutrients in the water column, despite the capacity of seagrass to uptake nutrients by their leaves (Viana et al., 2019b). For this reason, we state that competition is suspected but not stated in the literature.

**Table 1:** Measured traits in relation to the preemptable resource they are connected to.

**Trait category**	**Traits**	**Acronym**	**Scale**	**Unit**	**Description**	**Resource and references**	
						**Light**	**Inorganic nutrients**
Canopy forming traits	Leaf maximum length	Leaf ML	Leaf	cm	Distance between the end of the sheath and the tip of the longest leaf	1, 2, 3, 4, 5, 6, 7, 8, 9	Suspected, not stated in the literature
	Leaf maximum width	Leaf MW	Leaf	cm	Width of the broadest part of the leaf of the broadest leaf in a shoot.		
	Leaves/shoot	Leaves/Sh	Shoot	leaves shoot^−1^	Number of leaves in a shoot		
	Leaf mass area	LMA	Leaf	g cm^−2^	Grams of leaf tissue per unit of surface. See “Materials and methods” for further details.		
	Vertical rhizome length	VR length	Shoot	cm	Distance between the base of the shoot and the base of the sheath.		
	Shoots/meter	Shoots/M	Ramet	shoots m^−1^	Number of shoots of the ramet divided by the length of the ramet.		
Belowground structural traits	Rhizome diameter	RhD	Ramet	cm	Diameter of the cross section of the rhizome.	-	8, 9, 10, 11
	Roots/meter	Roots/M	Ramet	roots m^−1^	Number of roots of the ramet divided by the length of the ramet.		
	Root maximum length	Root ML	Ramet	cm	Vertical length of the longest root in the ramet.		

*All traits are continuous. References show seagrass studies suggesting a preemption effect of traits over the resources*.

*^1^Aleem (1955), ^2^Orth (1977), ^3^Zieman et al. (1984), ^4^Williams (1987), ^5^Williams (1990), ^6^Nomme and Harrison (1991b), ^7^Fourqurean et al. (1995), ^8^Vermaat et al. (1995), ^9^Duarte et al. (1998), ^10^Duarte et al. (2000), ^11^Ooi et al. (2011)*.

To quantify the seagrass traits at each site, we defined four zones of ~2,500 m^2^ per site, within which we sampled five ramets (defined as a train of at least two shoots) per species, amounting to a total of ~20 ramets per species and site. Ramets were carefully sampled using a shovel, avoiding leaves, shoot, and roots breakage. The ramets were transported frozen at −4°C to the ZMT for trait measuring. We measured leaf ML, leaf MW, VR length, RhD, and root ML with a ruler to the nearest millimeter, and leaves/sh, shoots/M, and roots/M were visually counted (as described in Table 1). For the measurement of the LMA (g cm^−2^), we took a subsample of the second leaf of one shoot per ramet. The subsample was cleaned of epiphytes and rinsed with distilled water and cut in squares for easier measurement of its surface with a ruler (cm^2^). It was then dried at 50°C in a forced air oven until constant DW. Finally, the LMA was calculated as the DW of the leaf subsample divided by its area (g DW cm^−2^).

The traits measured at the leaf and shoot levels (i.e., leaf ML, leaf MW, LMA, leaves/Sh, VR length) were averaged per ramet. This made a total of 573 ramets, distributed across eight seagrass species. The number of ramets per species were: *C. rotundata* (*n* = 61), *C. serrulata* (*n* = 74), *E. acoroides* (*n* = 44), *H. ovalis* (*n* = 83), *H. uninervis* (*n* = 81), *S. isoetifolium* (*n* = 50), *T. ciliatum* (*n* = 41), and *T. hemprichii* (*n* = 139) (see Supplementary Material 1 for the mean values of the measured traits per species per site).

### Data Analysis

We used R statistical software (R Core Team, 2020) for the analysis of the data. We made the plots with the package “ggplot2” (Wickham, 2016), with aesthetical changes done with the software InkScape (v. 2.0).

#### Clustering of Sites in Trophic States

The different trophic states of the seven sampling sites were identified according to macroalgae biomass, chlorophyll-*a* concentration in the water column, DIN and ${\text{PO}}_{4}^{3-}$ concentrations in the pore water, and δ^15^N in the sediment. A similarity matrix between sampling sites was constructed by means of a Euclidean distance matrix (Murtagh and Legendre, 2014), following a linear model criterion in order to minimize within-group sum of squares. From this matrix, classification of the sampling sites by trophic states based on the above-mentioned environmental variables was performed by Ward hierarchical clustering. In order to validate the membership of each sampling site to a specific cluster, we calculated the silhouette width index. This latter measurement is based on the comparison of the average dissimilarity between one sampling site and all the other sites within the cluster to which it belongs (cohesion), and the same measure computed for the closest cluster (separation). High silhouette width indexes (i.e., close to +1) in most clusters confirm the appropriate cluster configuration obtained (see silhouette plot in Supplementary Material 2).

#### Differences in Seagrass Cover Within and Among Trophic States

We investigated the seagrass community structure under two approaches, firstly, by assessing how the cover percentage of the different seagrass species varied within each trophic state (Model 1), and secondly, by assessing how the cover percentage of each seagrass species varied among the different trophic states in which the species were found (Model 2).

In order to perform both approaches, we used a generalized linear mixed-effects model with a beta distribution and logit link function, which is specifically adequate for proportional cover data. To build these models, we used the cover percentage of each seagrass species as the dependent variable, and sampling site and transect were set as random effects for both models. Seagrass species and trophic state were the categorical explanatory variables for Models 1 and 2, respectively. The only exception was the eutrophic state in Model 1, because it was composed of a single site, and therefore only transect was used as a random effect.

The models were validated for homoscedasticity, normality, and independence of the residuals. Model 1 showed problems with heteroscedasticity and residual dependence to transects. Model 2 showed some degree of heteroscedasticity and dependence to sampling transect for all species. In both cases we used a square root transformation to correct these validation problems. Significance of the categorical explanatory variables in both models was tested by analysis of variance (Type II test). We used the package “glmmTMB” (Brooks et al., 2017) for these analyses.

#### Differences in Probability of Space Preemption Within and Among Trophic States

In order to investigate whether the probability of space preemption of the seagrass species varied among sampling sites subjected to different trophic states, we built two different models. First, we studied the differences in probability of space preemption among seagrass species coexisting within the same trophic state (Model 3). Secondly, we studied if the probability of space preemption of each seagrass species varied among the trophic states (Model 4).

For both models, we used a generalized mixed-effects model with a binomial distribution and a logit link function, with probability of space preemption as the dependent variable, and sampling site and transect as random effects. We used seagrass species and the trophic state as the categorical explanatory variable for Models 3 and 4, respectively. The only exception was the eutrophic state in Model 3, because it was composed of a single site, and therefore only transect was used as a random effect.

The models were validated for homoscedasticity, normality, and independence of the residuals. Unfortunately, when Model 4 was applied to the species *C. rotundata, H. uninervis, S. isoetifolium*, and *T. hemprichii*, some level of heteroscedasticity and residual dependence to sampling site was shown and could not be resolved. Therefore, the results of these models should be conservatively interpreted. Significance of explanatory variables was tested with an analysis of variance (Type II test). We used the package “glmmTMB” (Brooks et al., 2017) for these analyses.

#### Characterization of the Functional Strategy (FS) of Seagrass Species for Interspecific Competition and Resource Preemption

Principal components analysis (PCA) enabled us to characterize the functional strategy of the different seagrass species based on the species trait values at each sampling site (Table 1). To run the PCA, species trait values were averaged for each species in each sampling site (Supplementary Material 1). We subsequently built a similarity matrix (Euclidean distance) between seagrass species by means of the seagrass standardized traits (Table 1). We obtained a single PCA where dots correspond to species and the number of dots per species represents the number of sites where the species occurred (*n* = 27). The scores of each dot in the principal components (PCs) of the PCA were, therefore, informative of the functional strategy of the seagrass species at the sampling sites. The first six PCs were retained for further analyses because these ensured a faithful representation of the initial functional dissimilarity among species within the ordination space (mean squared deviation = 0.000151) (Maire et al., 2015) (see Supplementary Material 3 for ordination diagnostics).

Additionally, we characterized the functional strategy of each seagrass species (i) across the study area and (ii) within each trophic state. To determine the functional strategy of a species across the study area, we calculated the centroid of the hypervolume enclosing all the species occurrences across sites in the six-dimensional space (i.e., the six PCs retained following Maire et al., 2015). Similarly, to identify the functional strategy of a species per trophic state, we calculated the centroid of the hypervolume enclosing all species occurrences in all sites within a trophic state in the six-dimensional space.

The correlations of each trait with the PCs, informative of the amount of the variability that is correlated to a given trait per PC, were then computed. We tested whether such correlations were significant by calculating their t-statistic (Yamamoto et al., 2014). We performed the PCA with the R packages “FactoMineR” (Le et al., 2008) and “factoextra” (Kassambara and Mundt, 2020).

#### Effect of the Trophic States on the Functional Strategy of Seagrass Species

To compare different functional strategies of a given seagrass species under different trophic states, we compared the centroids of the trophic states of each species in the PCA (see previous section). We used the coordinates of species' centroids in the oligotrophic state as a reference value. We subtracted this reference value to the coordinates of the centroids in the mesotrophic and eutrophic states. The value of this difference in each of the six PCs indicates the difference in the species' functional strategy under mesotrophic and eutrophic conditions.

#### Effect of the Seagrass Functional Strategy on Probability of Space Preemption

We further tested whether the functional strategy of a species has any effect on its probability of space preemption through the differences in the functional strategies of species pairs found together within plots (as seen in Figure 2).

For this purpose, we subtracted the species scores for every species pair. The scores of each dot in the PCs are informative of the functional strategy of the seagrass species at the sampling sites and, therefore, the difference indicates how similar or different the functional strategies between species pairs are. We followed Equation 3:


(3) $$\begin{array}{r} \Delta {FS}_{\;PC\;n}=Score_{SpeciesB}-Score_{SpeciesA} \end{array}$$

ΔFS _PCn_ is the difference in the functional strategy of a pair of species in PC n (n adopting a number between 1 and 6). Score _SpeciesB_ and Score _SpeciesA_ are the scores of each species in the PC n. ΔFS _PCn_ was then matched per species with the space preemption result as calculated in Equation 1.

Secondly, in order to test the effect of the ΔFS _PCn_ on the probability of pairwise space preemption (dependent variable), we fitted a generalized linear mixed-effects model with a binomial distribution and a logit link function. We created one model per PC conforming the functional strategy due to collinearity among several ΔFS _PCn_. We used ΔFS _PCn_, trophic state, and their interaction as the explanatory variables in each model. Site was included as a random effect to avoid confounding effects from other species present in the meadow and Species A and B to comply with the model validation assumptions.

In order to avoid spurious outcomes in the model we took three precautionary steps. First, we eliminated the eutrophic state from the dataset due to the presence of a unique species pair. Second, we did not use sampling transect as a random effect, as the species scores were calculated at the scale of site and not at the scale of transect. Third, we did not include any species pair present in less than five plots per site (see Supplementary Material 4 for the final number of pairwise interactions used). All models were validated for homoscedasticity, normality, and independence of the residuals. Final model selection and significance of explanatory variables were tested through model comparison (likelihood ratio test). We used the packages “lme4” for the construction of the generalized linear mixed-effects model (Bates et al., 2015) and “lmtest” for the model selection (Zeileis and Hothorn, 2002).

#### Prediction of Probability of Preemption by PC1 of the Functional Strategy of Seagrass

From the previous analyses we identified PC1 as the main driver for space preemption between species pairs growing together in the same plot. Therefore, in a further step, we aimed to predict the probability of space preemption (as calculated in Equation 2) by a seagrass species due to its score on PC1 as a proxy of its functional strategy.

For this purpose, we used a generalized linear mixed-effects model with a binomial distribution and a logit link function. Species' scores on PC1 were used as a fixed effect in the model. Site and seagrass species were included as a random effect to avoid confounding effects from other species present in the meadow and to comply with the model validation assumptions, respectively. Significance of explanatory variables was tested through model comparison (likelihood ratio test). All models were validated for homoscedasticity, normality, and independence of the residuals.

We used the packages “lme4” for the construction of the generalized linear mixed model (Bates et al., 2015) and “lmtest” for the model selection (Zeileis and Hothorn, 2002).

## Results

### Trophic States and Indicators

**Figure 4:**
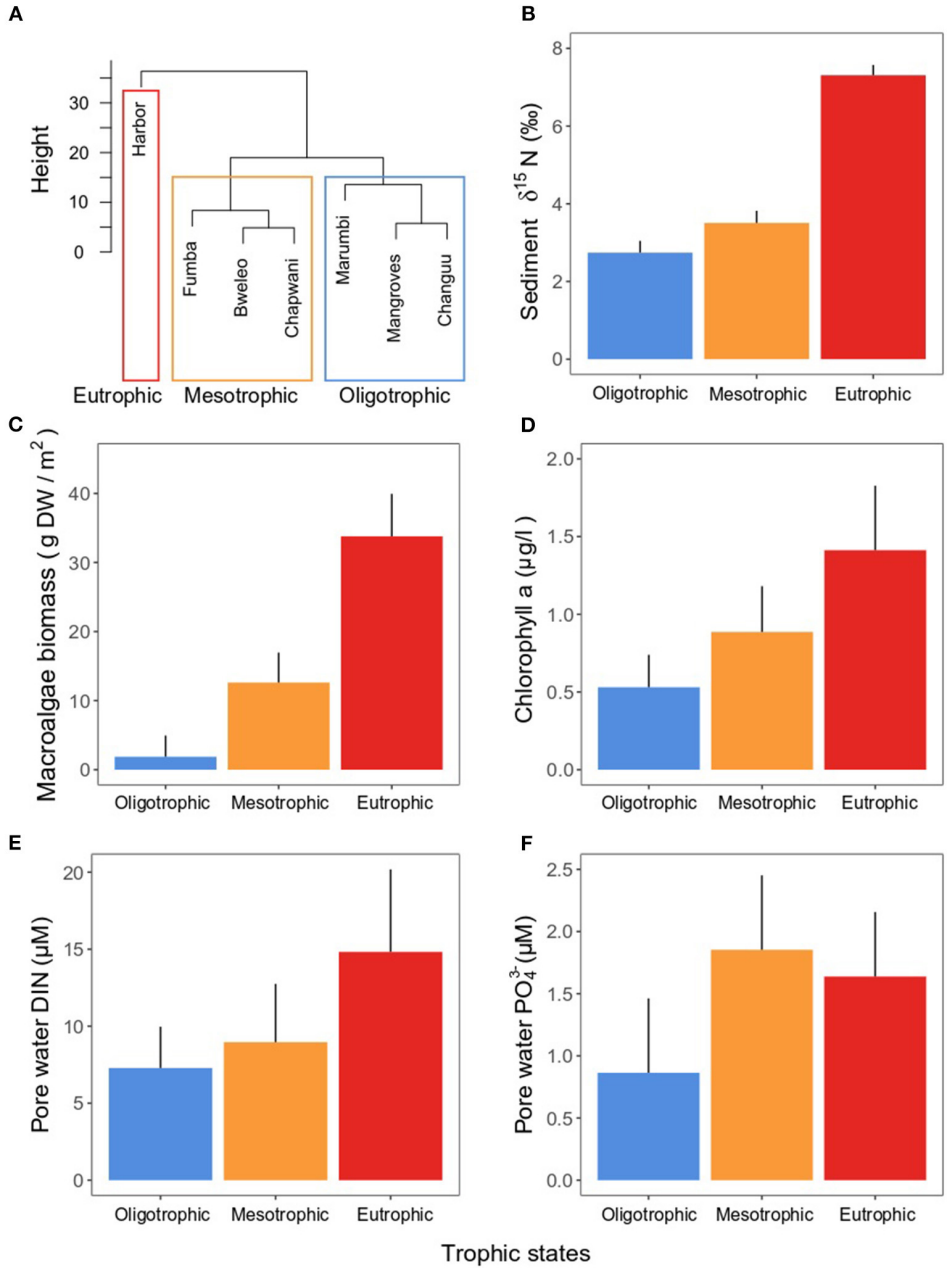
**(A)** Ward clustering of sites according to trophic state. Mean (± SE) **(B**) sediment δ^15^N, **(C)** macroalgae biomass, **(D)** chlorophyll-*a* in the water column, **(E)** pore water DIN, and **(F)** pore water ${\text{PO}}_{4}^{3-}$, taken as proxies for eutrophication indicators. Means were calculated among samples taken in the sites (three sites for the oligotrophic and mesotrophic states, one site for the eutrophic state). For macroalgae biomass, *n* = 45 for the oligo- and mesotrophic states, and *n* = 15 for the eutrophic state. For δ^15^N, chlorophyll-*a*, pore water DIN, and pore water ${\text{PO}}_{4}^{3-}$, *n* = 15 for the oligo- and mesotrophic states, and *n* = 5 for the eutrophic state.

Sites clustered in two groups marking areas subject to oligotrophic and mesotrophic conditions, whereas a single site was markedly different from all others given its high eutrophication (Figures 1, 4A). δ^15^N in the sediment was double in the eutrophic site than in the oligo- and mesotrophic areas (Figure 4B), indicating discharge of human waste water in the eutrophic site. These differences were also reflected in the macroalgae biomass. While the oligotrophic sites had a macroalgae biomass close to zero (mean ± SE: 1.87 ± 3.07 g DW m^−2^), the mesotrophic (12.61 ± 4.35 g DW m^−2^) and eutrophic (33.78 ± 6.15 g DW m^−2^) sites had nearly 7 and 18 times the biomass of the oligotrophic site, respectively (Figure 4C). Similarly, the concentration of chlorophyll-*a* in the water column (oligotrophic: 0.53 ± 021 < mesotrophic: 0.88 ± 0.29 < eutrophic: 1.41 ± 0.41 μg l^−1^) and DIN in pore water (oligotrophic: 7.28±2.68 < mesotrophic: 8.96 ± 3.79 < eutrophic: 14.82 ± 5.36 μM) steadily increased along the trophic states (Figures 4D,E). Lastly, the concentration of ${\text{PO}}_{4}^{3-}$ in the pore water reached its maximum in the mesotrophic sites (1.85 ± 0.60 μM), being slightly lower in the eutrophic site (1.63 ± 0.51 μM) and approximately half in the oligotrophic sites (0.86 ± 0.60 μM) (Figure 4F).

### Seagrass Cover and Probability of Preemption Across Trophic States

**Figure 5:**
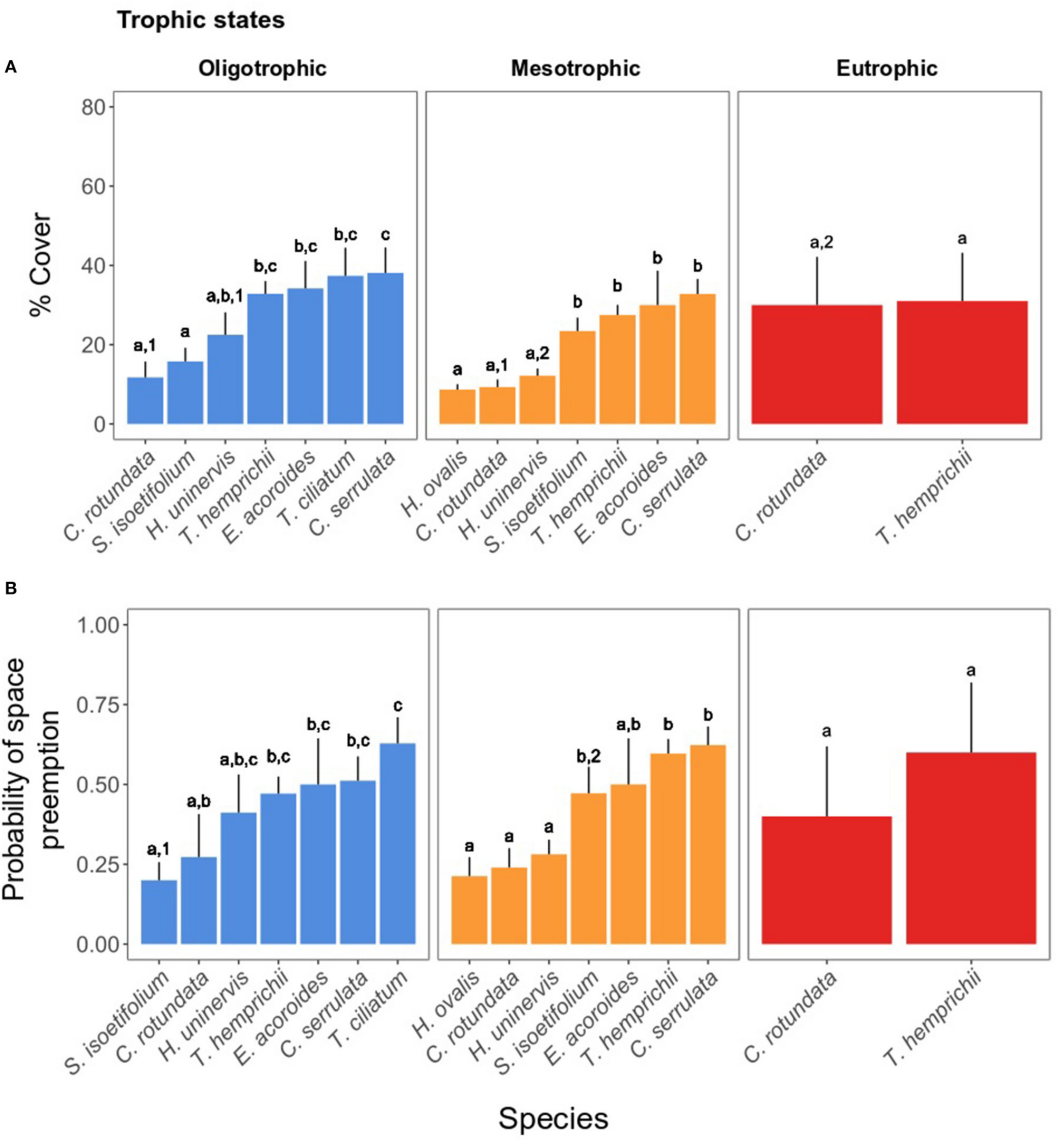
**(A)** Seagrass cover % of each species in the trophic states. **(B)** Probability of preemption of the seagrass species in the trophic states. The letters indicate significant differences within the states and among species, whereas the numbers indicate significant differences within a species among states. For the statistical output, see Tables 2, 3. For the statistical output of pairwise differences, see Supplementary Material 5.

When present, the opportunistic *C. serrulata* and the climax species *E. acoroides, T. ciliatum*, and *T. hemprichii* were dominant in coverage (> 27%) regardless of the trophic state (Figure 5A). The pioneer *C. rotundata, H. uninervis*, and *H. ovalis* were the least abundant (8–30%) throughout the study area. *S. isoetifolium* was among the least abundant species under oligotrophic conditions (mean ± SE: 15.77 ± 3.43%), yet equally abundant to climax species under a mesotrophic regime (Figure 5A and Table 2). The opposite occurred for *H. uninervis*, which was more abundant in the oligotrophic (22.50 ± 5.65%) than the mesotrophic sites (12.16 ± 1.83%). The seagrass meadows in the eutrophic site were composed of only two seagrass species, *T. hemprichii* and *C. rotundata*. The latter was the only species showing significant differences in coverage among the three trophic states (Table 2), reaching at the eutrophic site twice the coverage it reached in areas of oligo- and mesotrophic conditions (eutrophic: 30.00 ± 12.14% > mesotrophic: 9.31 ± 1.91% ≈ oligotrophic: 11.75 ± 4.02%), making its coverage comparable to that of *T. hemprichii*.

**Table 2:** Complete output of Models 1 and 2 assessing (a) Model 1: Differences in cover % of the seagrass species within trophic states and (b) Model 2: Differences in cover % of each seagrass species across trophic states.

**Response variable: Seagrass cover %**	**Explanatory variables**	**χ^2^**	**df**	***P*** **-value**
	**Random effects**		**Variance**	**Std deviation**
**a) Model 1: Differences in cover % of the seagrass species within trophic states**				
Oligotrophic	**Species**	**24.085**	**6**	**0.0005**
	Random effects	Site	7.06·10^−13^	8.40·10^−7^
		Transect	3.34·10^−11^	5.78·10^−6^
Mesotrophic	**Species**	**62.871**	**6**	**1.173·10** ^**−11**^
	Random effects	Site	0.0015	0.1244
		Transect	2.51·10^−9^	5.02·10^−5^
Eutrophic	Species	0.0014	1	0.9699
	Random effects	Transect	4.59·10^−11^	6.78·10^−6^
**b) Model 2: Differences in cover % of each seagrass species across trophic states**				
*C. rotundata*	**Trophic state**	**9.511**	**2**	**0.0086**
	Random effects	Site	8.60·10^−13^	9.27·10^−7^
		Transect	1.73·10^−10^	1.31·10^−5^
*C. serrulata*	Trophic state	0.014	1	0.9044
	Random effects	Site	5.23·10^−10^	2.28·10^−5^
		Transect	0.2879	0.5366
*E. acoroides*	Trophic state	0.1749	1	0.6758
	Random effects	Site	1.26·10^−14^	1.12·10^−7^
		Transect	2.24·10^−10^	1.49·10^−5^
*H. uninervis*	**Trophic state**	**3.928**	**1**	**0.0474**
	Random effects	Site	1.91·10^−10^	1.38·10^−5^
		Transect	0.1030	0.3209
*S. isoetifolium*	Trophic state	0.377	1	0.5389
	Random effects	Site	3.33·10^−10^	1.82·10^−5^
		Transect	0.3182	0.5641
*T. hemprichii*	Trophic state	0.562	2	0.755
	Random effects	Site	0.1066	0.3265
		Transect	0.0134	0.1158

*Significance is highlighted in boldface (p < 0.05). Std deviation, standard deviation*.

The probability of preemption of a given seagrass species (Table 3 and Figure 5B) across trophic states mirrored the spatial patterns of cover percentage. Climax species, together with *C. serrulata*, tended to have the highest probability of preemption regardless of the trophic state. *S. isoetifolium* was the only pioneer species to be more likely to preempt space under mesotrophic conditions (0.47 ± 0.08) in comparison to oligotrophic conditions (0.20 ± 0.05).

**Table 3:** Complete output of models assessing (a) Model 3: Differences in probability of space preemption of the seagrass species within trophic states and (b) Model 4: Differences in the probability of space preemption of each seagrass species across trophic states.

**Response variable: Probability of preemption**	**Explanatory variables**	**χ^2^**	**df**	***P*** **-value**
	**Random effects**		**Variance**	**Std deviation**
**a) Model 3: Differences in probability of space preemption of the seagrass species within trophic states**				
Oligotrophic	**Species**	**17.916**	**6**	**0.0064**
	Random effects	Site	7.87·10^−13^	8.87·10^−7^
		Transect	5.17·10^−10^	2.27·10^−5^
Mesotrophic	**Species**	**46.224**	**6**	**2.67·10^−8^**
	Random effects	Site	9.93·10^−11^	9.96·10^−6^
		Transect	4.54·10^−10^	2.13·10^−5^
Eutrophic	Species	0.3946	1	0.5299
	Random effects	Transect	2.37·10^−10^	1.54·10^−5^
**b) Model 4: Differences in the probability of space preemption of each seagrass species across the trophic states**				
*C. rotundata*	Trophic state	0.605	2	0.7389
	Random effect	Site	2.64·10^−9^	5.14·10^−5^
*C. serrulata*	Trophic state	1.346	1	0.2458
	Random effect	Site	1.89·10^−10^	1.37·10^−5^
*E. acoroides*	Trophic state	0.000	1	1.0000
	Random effect	Site	2.72·10^−10^	1.65·10^−5^
*H. uninervis*	Trophic state	1.149	1	0.2838
	Random effect	Site	2.038·10^−10^	1.42·10^−5^
*S. isoetifolium*	**Trophic state**	**6.875**	**1**	**0.0087**
	Random effect	Site	6.64·10^−10^	2.57·10^−5^
*T. hemprichii*	Trophic state	0.393	2	0.8214
	Random effect	Site	0.4266	0.6531

*Significance is highlighted in boldface (p < 0.05). Std deviation, standard deviation*.

### Functional Strategy of Seagrass Species as Defined by Their Traits

**Figure 6:**
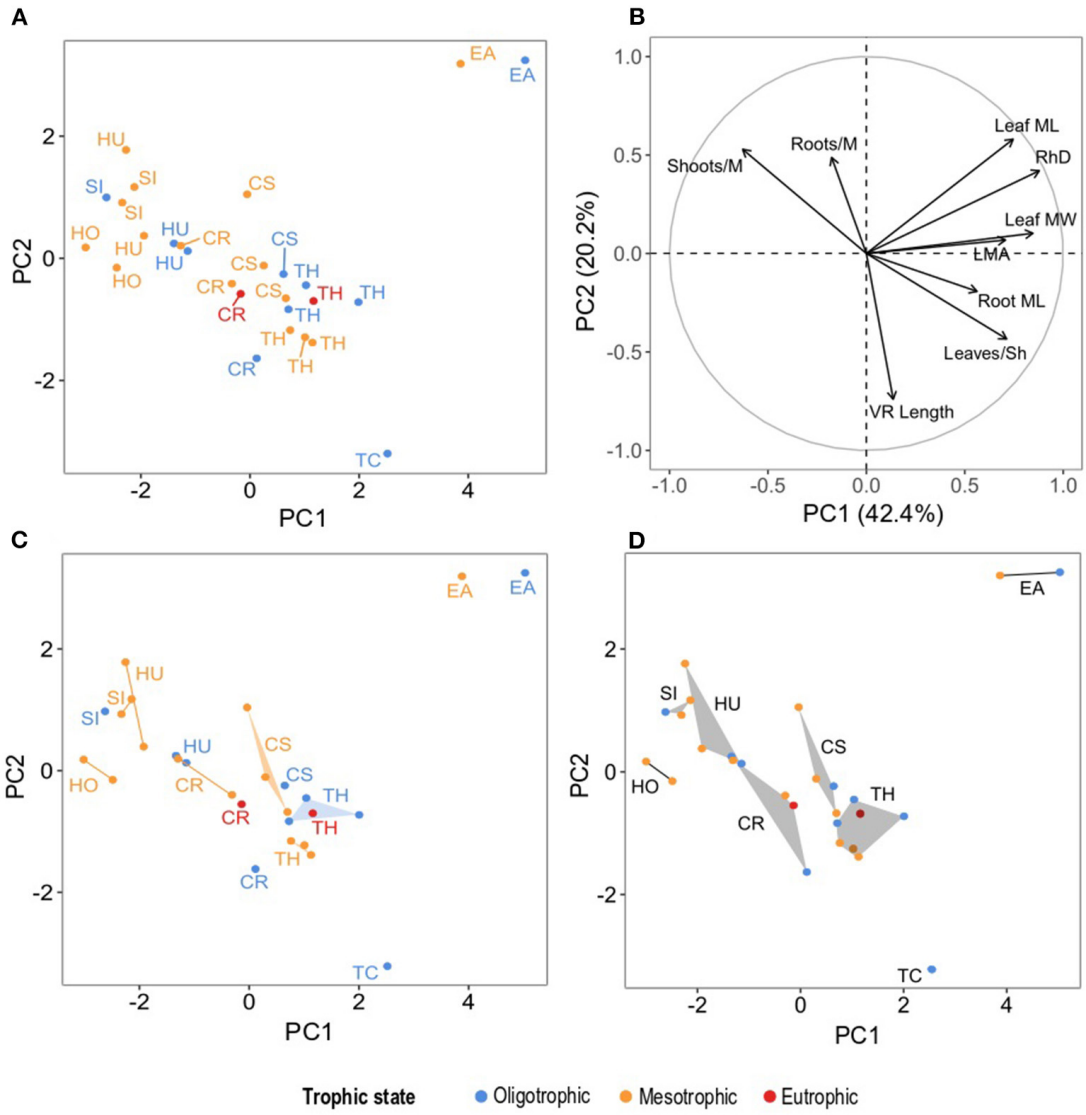
Principal components analysis, **(A)** dots correspond to seagrass species per site plotted on PC1 and PC2 (which collectively explained 62.6% of the unconstrained variability), **(B)** traits as vectors and their correlation with PC1 and PC2 (Table 5 for trait nomenclature and PCA output), **(C)** species' hypervolumes per trophic state projected on PC1 and PC2, **(D)** species' hypervolumes across all study sites projected on PC1 and PC2. For the graphical representation of PCs 3-6 see Supplementary Material 6: Section 4. Colors represent trophic states and acronyms in **(A,C,D)** represent species' names abbreviated as follows: CR, *C. rotundata*; CS, *C. serrulata;* EA, *E. acoroides*; HO, *H. ovalis*; HU, *H. uninervis*; SI, *S. isoetifolium*; TC, *T. ciliatum*; TH, *T. hemprichii*.

The functional traits included in the ordination reflected the differential strategies that seagrass take regarding interspecific competition across sites as reflected by the species centroids. PC1 explained 43.46% of the unconstrained variability in the PCA and it was determined primarily by their RhD (0.88) and leaf MW (0.85) (Figures 6A–D and Tables 4, 5). Along PC1 the distribution of seagrass species centroids marked a size gradient, from the smallest ephemeral species to the two larger climax species: *H. ovalis, S. isoetifolium, H. uninervis, C. rotundata, C. serrulata, T. hemprichii, T. ciliatum*, and *E. acoroides*. This PC also reflected the correlation among traits indicative of size of plant structures (aforementioned traits, together with leaf ML, leaves/Sh, LMA, root ML), indicating that they all increase and decrease in size collectively, both for canopy forming and belowground traits. Interestingly, the trait defining the density of shoots (shoots/M) was inversely correlated to PC1. This suggests a trade-off between the size of the seagrass plant and shoot density of seagrass.

**Table 4:** Centroids of the species hypervolumes in the six dimensions of the multidimensional space created by the ordination of the functional traits (Figure 6).

**Species**	**PC 1**	**PC 2**	**PC 3**	**PC 4**	**PC 5**	**PC 6**
*C. rotundata* (CR)	−0.40	−0.60	−0.04	−0.40	−0.86	0.43
*C. serrulata* (CS)	0.40	0.00	0.79	−0.98	−0.14	−0.90
*E. acoroides* (EA)	4.45	3.22	−0.32	−0.54	0.25	0.45
*H. ovalis* (HO)	−2.75	0.01	−1.34	−1.27	1.59	0.21
*H. uninervis* (HU)	−1.67	0.64	0.71	1.17	−0.04	0.05
*S. isoetifolium* (SI)	−2.36	1.03	0.16	−0.40	−0.45	0.29
*T. ciliatum* (TC)	2.52	−3.21	2.74	−0.97	1.07	0.13
*T. hemprichii* (TH)	1.12	−0.93	−0.82	0.95	0.11	−0.09

**Table 5:** Correlations of the functional traits to the dimensions of the multidimensional space (Figure 6).

	**PC 1**	**PC 2**	**PC 3**	**PC 4**	**PC 5**	**PC 6**
	**43.36%**	**20.22%**	**12.42%**	**11.29%**	**5.62%**	**4.62%**
**Canopy forming traits**						
Leaf maximum length (leaf ML)	**0.75**	**0.58**	−0.02	−0.10	−0.04	0.29
Leaf maximum width (leaf MW)	**0.85**	0.10	−0.01	−0.28	0.36	−0.13
Leaves/shoot (leaves/Sh)	**0.71**	**−0.43**	0.19	0.28	0.27	−0.25
Leaf mass area (LMA)	**0.71**	0.07	−0.14	**0.61**	−0.25	0.09
Vertical rhizome length (VR length)	0.14	**−0.74**	**0.49**	−0.02	0.10	**0.43**
Shoots/meter (shoots/M)	**−0.63**	**0.53**	**0.41**	−0.20	0.13	0.05
**Belowground structure traits**						
Rhizome diameter (RhD)	**0.88**	**0.42**	0.03	−0.14	0.06	0.09
Roots/meter (roots/M)	−0.18	**0.49**	**0.67**	**0.48**	0.03	−0.10
Root maximum length (root ML)	**0.60**	−0.19	**0.46**	**0.42**	**−0.46**	−0.21

*Numbers in boldface and underlined indicate a trait with a significant correlation to the dimension (p < 0.05). For statistical output of the trait correlation, see Supplementary Material 6: Section 2*.

In PC2 (20.22%), all the seagrass species except *T. ciliatum* and *E. acoroides* were somewhat grouped, showing that this dimension separated the two climax species (Tables 4, 5 and Figures 6A–D). Despite both the species showing a similar centroid coordinate in PC1, this was achieved with different traits according to PC2. *T. ciliatum* showed higher VR length and leaves/Sh, whereas *E. acoroides* showed higher leaf ML, RhD, and density of roots (roots/M) and shoots (shoots/M). In PC3 (12.42%) (Tables 4, 5), species' scores mainly reflected variability in roots/M. In this dimension, *H. ovalis* showed an inversed correlation to this and other traits (VR length, shoots/M, root ML), indicating that it had a different functional strategy than the rest of the pioneer seagrass species, with not only small plant size, but also with sparse root and shoot density. For the report on unconstrained variability explained by all PCs, trait correlations and seagrass species centroids of PCs 4, 5, and 6, please see Supplementary Material 6.

### Effect of the Trophic State on the Functional Strategy of Seagrass Species

**Figure 7:**
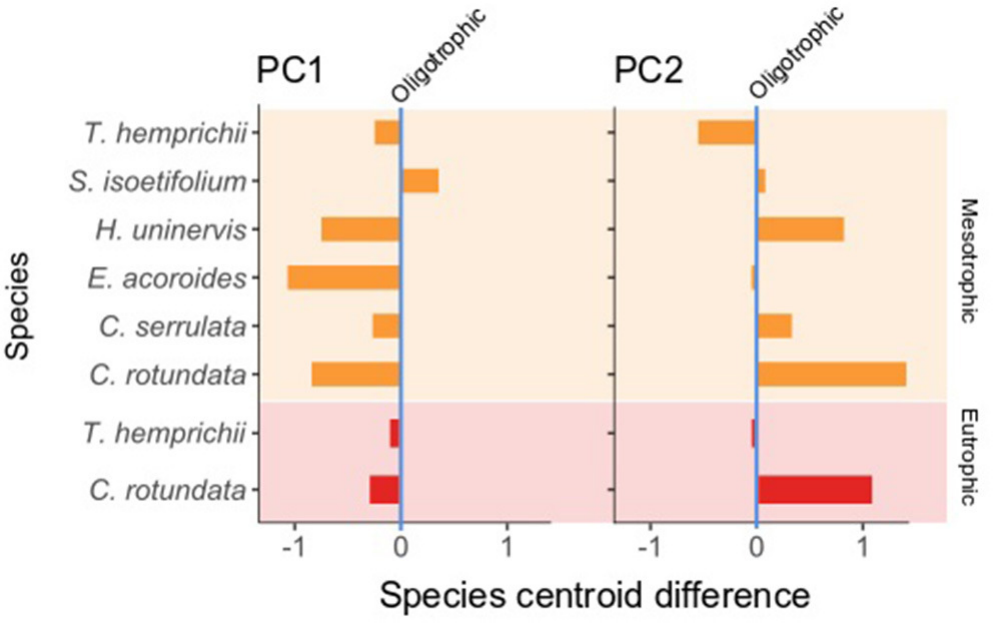
Difference in the species centroids from the oligotrophic (vertical zero axis) to the mesotrophic (orange bar, light orange background) and eutrophic (red bars, light red background) states. *T. ciliatum* and *H. ovalis* are not present in these plots due to their presence uniquely under a single trophic state, oligotrophic and mesotrophic, respectively. For the differences in species centroids in the rest of the PCs see Supplementary Material 7.

The trophic state had different effects for each seagrass species and dimension (PC) of their functional strategy. This was reflected by the difference in the species centroid in the oligotrophic, mesotrophic, and eutrophic states (Figures 6C, 7). Between the oligotrophic and mesotrophic states, we saw both general and species-specific responses in the functional strategy of seagrass.

Species' centroids on PC1 were lower under mesotrophic than under oligotrophic conditions (Figure 7). This indicated a general decrease in seagrass plants size (leaf ML and MW, RhD, root ML, leaves/Sh) and an increase in shoots/M, suggesting that a functional strategy more typical of opportunistic/pioneer species was selected for mesotrophic conditions. The only exception was *S. isoetifolium*, whose centroid was higher in mesotrophic sites. The same trend was true in PC1 in the differences in the centroids of *C. rotundata* and *T. hemprichii* between the oligotrophic and eutrophic states. In PC2 (Figure 7), all species except *T. hemprichii* showed very low or positive differences in their centroids between oligotrophic and mesotrophic conditions. VR length was inversely correlated to this PC (Table 5 and Figure 6C), suggesting that an increase in vertical rhizome length was not a selected functional strategy under the mesotrophic state.

Due to the low amount of unconstrained variability explained by the rest of the PCs (PC3 = 12.42%, PC4 = 11.29%, PC5 = 5.62%, PC6 = 4.62%) the centroid migrations among trophic states were not reported in depth (see Supplementary Material 6 for the results).

### Difference in the Seagrass Functional Strategy and the Probability of Space Preemption

**Figure 8:**
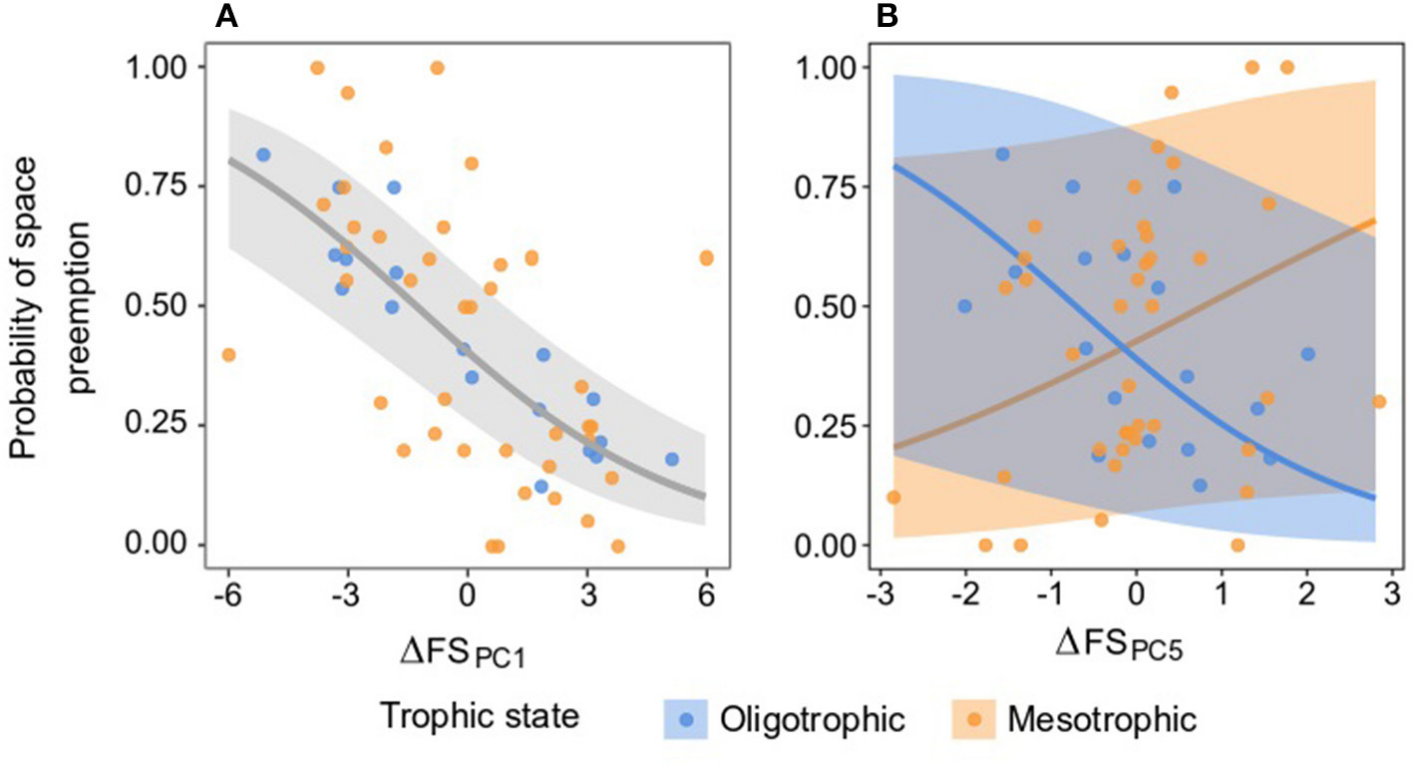
Relationship between the difference in the functional strategy of a pair of species on PCs 1 **(A)** and 5 **(B)** (ΔFS _PC1,5_) on the probability of space preemption of species A on species B. The points represent the empirical space preemption for each seagrass species pair. **(A)** The gray line represents the predicted preemption according to ΔFS _PC1_ and the light gray ribbons the confidence interval of the prediction. Model equation for ΔFS _PC1_: $\ln\left(\frac{p_{i}}{1-p_{i}}\right)=-0.391-0.301•\Delta FSPC1$. **(B)** The blue and orange lines represent the predicted preemption per trophic state according to ΔFS _PC5_, and the light blue and orange ribbons the confidence intervals of the prediction. See Supplementary Material 8 for the regressions in the other PCs.

The probability of space preemption was only significantly explained by ΔFS _PC1_ (Table 6 and Figure 8A). The effect of this dimension of the functional strategy on the probability of space preemption did not differ between trophic states, indicating that regardless of the trophic state, the same traits were responsible for preemption. PC1 correlated positively with traits related to plant size and negatively with shoots/M (Table 5). The probability of Species A preempting space from Species B was highest when the score of Species A on PC1 was higher than the score of Species B on PC1 (i.e., ΔFS_PC1_ < 0). Conversely, the probability of Species B preempting space from Species A was highest when Species B scored higher on PC1 than Species A (ΔFS _PC1_ > 0). When Species A and B scored similarly on PC1 (ΔFS_PC1_ ≈ 0), both species had equal probability (50%) of preempting space from the other (Figure 8A).

**Table 6:** Complete output of generalized linear mixed-effects model testing the effect of the difference in the functional strategies (ΔFS) of a seagrass pair on the probability of space preemption under different trophic states.

**Response variable: Probability of space preemption**	**Explanatory variables**	**χ^2^**	**df**	***P*** **-value**
	**Random effects**		**Variance**	**Std deviation**
PC 1	**ΔFS**_**PC1**_	**19.7010**	**1**	**9.06·10** ^**−6**^
	Trophic state	0.5040	1	0.4778
	ΔFS _PC1_: Trophic state	0.0017	1	0.9675
	Random effects	Site	0.0061	0.0777
		Species A	0.1290	0.3592
		Species B	0.0140	0.1183
PC 2	ΔFS _PC2_	2.5085	1	0.1132
	Trophic state	0.3826	1	0.5362
	ΔFS _PC2_: Trophic state	0.7890	1	0.3744
	Random effects	Site	0.0122	0.1105
		Species A	0.6443	0.8027
		Species B	0.3682	0.6068
PC 3	ΔFS _PC3_	0.1641	1	0.6854
	Trophic state	0.4836	1	0.4867
	ΔFS _PC3_: Trophic state	3.5475	1	0.0596
	Random effects	Site	0.0138	0.1177
		Species A	0.7500	0.8660
		Species B	0.4712	0.6865
PC 4	ΔFS _PC4_	0.3407	1	0.5595
	Trophic state	0.4594	1	0.4979
	ΔFS _PC4_: Trophic state	0.5307	1	0.4663
	Random effects	Site	0.0134	0.6448
		Species A	0.6758	0.8221
		Species B	0.4157	0.6448
PC 5	ΔFS _PC5_	0.0058	1	0.9394
	Trophic state	0.4687	1	0.4935
	**ΔFS**_**PC5**_**: Trophic state**	**9.6665**	**1**	**0.0018**
	Random effects	Site	0.0067	0.0820
		Species A	0.4857	0.6969
		Species B	0.2829	0.5319
PC 6	ΔFS _PC6_	2.1849	1	0.1394
	Trophic state	0.7199	1	0.3962
	ΔFS _PC6_: Trophic state	1.7076	1	0.1913
	Random effects	Site	0.0123	0.1109
		Species A	0.6953	0.8338
		Species B	0.4147	0.6440

*Significance is highlighted in boldface (p < 0.05). Std deviation, standard deviation*.

Differences in species scores on PC2, PC3, PC4, and PC6 played a negligible role in determining the probability of space preemption. Interestingly, the role of ΔFS on PC5 in driving space preemption was contingent on the trophic state (Table 5). ΔFS _PC5_ was inversely related to the probability of pairwise space preemption under oligotrophic, yet positively related to the probability of preemption under mesotrophic conditions (Figure 8B). However, the lack of significance of PC5 as a fixed effect (χ^2^ = 0.005, df = 1, *p* = 0.939) and the high confidence intervals (approximately double of the fitted values) make it difficult to make inferences from this result.

### Probability of Space Preemption of Seagrass Species as Determined by PC1

**Figure 9:**
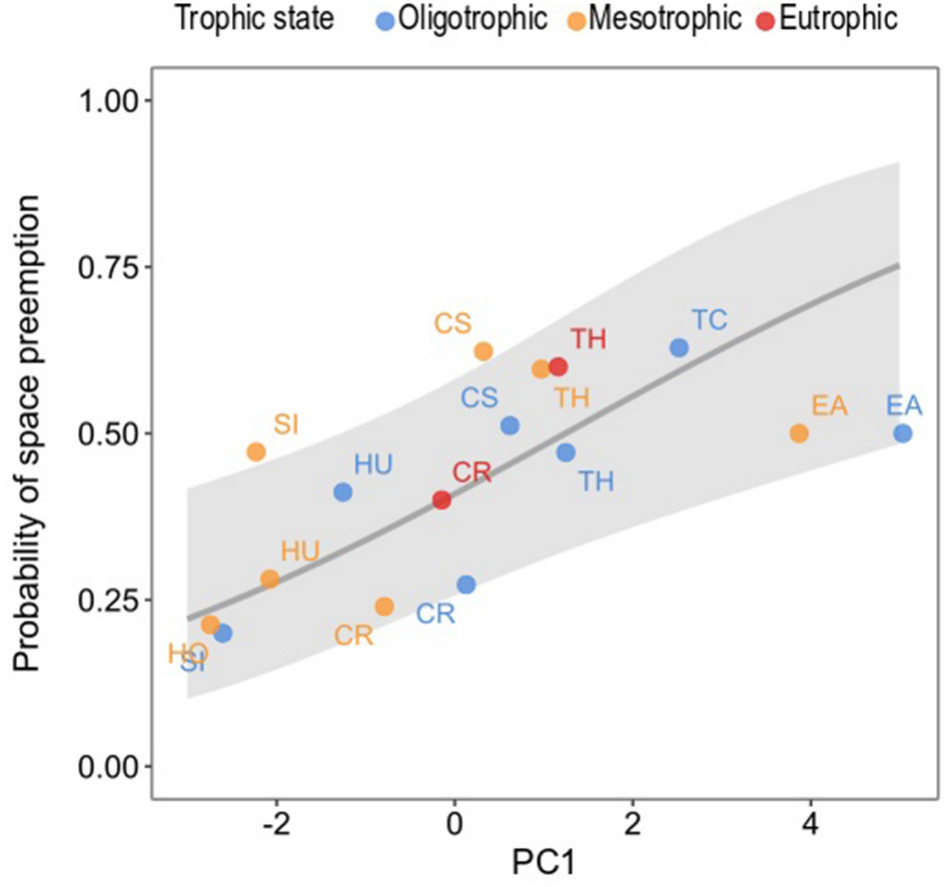
Probability of preemption of all seagrass species present in the studied meadows in Unguja Island as explained by their scores in PC1. Each data point is the score of the seagrass species in a trophic state, coded by their corresponding colors, and the letters are the seagrass species. The gray line is the fitted line and the gray ribbons the confidence intervals of the prediction of preemption. Model equation: $\ln\left(\frac{p_{i}}{1-p_{i}}\right)=-0.367+0.296PC1$. Seagrass species: CR, *C. rotundata*; CS, *C. serrulata;* EA, *E. acoroides*; HO, *H. ovalis*; HU, *H. uninervis*; SI, *S. isoetifolium*; TC, *T. ciliatum*; TH, *T. hemprichii*.

As previously explained, ΔFS _PC1_ was identified as the driver behind space preemption in seagrass species pairs and we used it to predict the probability of preemption of each seagrass species as a result of their scores in this dimension (Figure 9). The probability of space preemption by a given seagrass species significantly increased with its score on PC1 (χ^2^ = 7.796, df = 1, *p* = 0.005). In other words, the greater the size of a seagrass species (as defined by leaf MW, ML, RhD, root ML, LMA), the higher the likelihood it would preempt space from other species.

## Discussion

Tropical seagrass meadows offer unique opportunities for the study of species interactions due to the high diversity of species sharing the same habitat (Short et al., 2007). This is the case of Unguja Island, located in the Western Indian Ocean, one of the hotspots of seagrass biodiversity, in which we found meadows occupied by an array of different seagrasses with a variety of life-history strategies. Competition for space is an ecological process common for all sessile organisms, and has been widely reported both in terrestrial plants (meta-analysis on the topic by Kinlock, 2019) and seagrass (Aleem, 1955; Phillips, 1960; den Hartog, 1970; Birch and Birch, 1984; Williams, 1990; Duarte et al., 2000; Willette and Ambrose, 2012). However, a description of the potential mechanisms behind the preemption for space has been generally suggested and not tested in seagrass (Orth, 1977; Zieman et al., 1984; Williams, 1987; Nomme and Harrison, 1991b; Fourqurean et al., 1995; Vermaat et al., 1995; Duarte et al., 1998; Ooi et al., 2011). This study related, for the first time, seagrass morphological traits with their capacity to exert space preemption on other seagrass species. Specifically, we identified a positive correlation between bigger structures (leaf ML, leaf MW, RhD, root ML, among others) and probability of space preemption.

### A Trait-Based Approach to Differentiate the Functional Strategy of Seagrass Species

Seagrass species have been classified according to different criteria, generally in a categorical fashion differentiating pioneer, opportunistic, and climax species. These classifications prove useful when discussing succession in the development of a meadow (Williams, 1990) and in the differentiation between transitory and persistent meadows (Kilminster et al., 2015). However, they generally provide little insight in other ecological processes and functions. It is for this reason that trait-based approaches (TBAs) are considered fundamental tools that may be used to better understand how traits of organisms affect their response to environmental drivers (Viana et al., 2020) and in turn how these traits affect ecosystem functions and processes (Gustafsson and Norkko, 2016; Jänes et al., 2017). With TBAs the existing body of knowledge can be directly used to select biologically relevant functional traits linked to the research question at hand (Violle et al., 2007; Mouillot et al., 2013). In the present study, we were able to order seagrass species in a continuous fashion using traits related to resource preemption. What this allows is the creation of scores per species in the multidimensional space that have a quantifiable biological meaning, an approach that sets it apart from traditional categorical classifications. In PC1, species centroids were ordered in a density to size gradient. Smaller species, like *H. ovalis, S. isoetifolium, H. uninervis*, and *C. rotundata* had negative scores, while *T. hemprichii, T. ciliatum*, and *E. acoroides* had positive scores. Interestingly, *C. serrulata* had a positive centroid near zero, showing a mixed strategy between the two groups. This showed a trade-off from density to size that had important implications when it came to space preemption as discussed in the next sections.

TBAs, therefore, offer the opportunity to directly link seagrass functional traits to the ecological process of interest for the study. It is, however, of fundamental importance to use traits that have been reported to affect the process at hand to obtain a multidimensional space of biological meaning.

### Response of Seagrass Functional Strategy to Different Trophic States

Seagrass meadows in Unguja Island were subjected to varying trophic states ranging from oligotrophic to eutrophic, albeit only in one site. While seagrass meadows were composed of seven species in the oligotrophic and mesotrophic sites, only two species were found in the eutrophic site, *C. rotundata* and *T. hemprichii*. The final species arrangement for each site was controlled not only by species competition but also by two other filters, namely species dispersion and the abiotic filter (Tilman, 1985; Keddy, 1992; Garnier et al., 2016). Although we did not study the effect of these two filters on the presence/absence of seagrass in different sites, it was possible to detect changes in the functional traits among the trophic states.

Seagrass traits are good indicators of change and stress (Roca et al., 2016), and can give insights on the effect of the environmental conditions on the seagrass species. The traits selected for this study were not specifically chosen as response traits, but rather due to their functional relationship with resource preemption. However, the traits selected were morphological, which have been reported to change under varying environmental conditions (Mvungi and Pillay, 2019; Artika et al., 2020; Viana et al., 2020). This was also the case in this study, in which seagrass species revealed differences in their centroids in PC1 under different trophic states. All species with the exception of *S. isoetifolium* showed a decrease in the value of the coordinates of their centroids in PC1, which translates in a decrease in size and an increase in shoot density.

Generally, in nutrient-limited systems, a higher concentration of inorganic nutrients (both nitrogen and phosphorus) in the environment correlates with an increase in seagrass growth. *T. hemprichii* and *C. rotundata* have increased leaf growth and biomass under nutrient enrichment (Agawin et al., 1996; Terrados et al., 1999). Similarly, *H. uninervis* and *S. isoetifolium* have been found to increase their growth rate under nutrient enrichment, in addition to changes in biochemical traits (Udy et al., 1999). However, eutrophication encompasses other environmental conditions apart from an increase in nutrients (Burkholder et al., 2007). Indirect consequences of eutrophication include light attenuation due to the growth of epiphytes, macroalgae and phytoplankton, and anoxic sediments, with associated negative consequences for seagrasses. *T. hemprichii* has responded to shading by showing morphological stress symptoms, like reduced shoot growth and lower belowground biomass (Browne et al., 2017), and the production of new, altered leaves with reduced length, width, and thickness (Collier et al., 2012). This response is shared by *C. serrulata* and *H. uninervis* (Collier et al., 2012), despite other reports indicating an increase in vertical rhizome length for *C. serrulata* under shading (Lam et al., 2004). These results agree with the general trend of seagrass in the present study showing lower values for their centroids in PC1 and, consequently, a reduction in size under mesotrophic conditions. A similar trend is shared in the difference in the centroids of *T. hemprichii* and *C. rotundata* between oligotrophic and eutrophic conditions. The only species in this study increasing the value of its centroid under mesotrophic conditions, *S. isoetifolium*, has also been reported to increase its leaf growth and elongation under shading (Fokeera-Wahedally and Bhikajee, 2005).

These results indicate a certain amount of species-level plasticity, allowing morphological traits to undergo changes potentially aiding species survival under different environmental conditions. Additionally, the change in species morphological traits has consequences in the probability of space preemption among seagrass species.

### Seagrass Functional Strategy and Its Control on Space Preemption

Competition between plants has been described as an asymmetric phenomenon (Schwinning and Weiner, 1998). This means that, while one plant can exert a negative effect on a second plant, there is usually not a reciprocal effect (Davis and Fourqurean, 2001). The reason behind this asymmetry has been generally related to plant size, but it depends on the resource type. In the case of light, as a directional resource, preemption is directly related to size of plant structures (Weiner and Fishman, 1994; Horvitz and Schemske, 2002), i.e., how much light a plant can block from smaller plants. However, the case of inorganic nutrients is more complicated (Schwinning and Weiner, 1998) and depends on the plant nutritional demands (Fourqurean et al., 1995), uptake capacity, and the nutrient distribution in the sediment. Additionally, submerged marine plants can obtain nutrients from the water column (Viana et al., 2019b), thus complicating the identification of traits related to nutrient preemption. This is, likely, one of the reasons why we could not find studies directly addressing the relation between aboveground seagrass traits and inorganic nutrients preemption (Table 1). Our study showed that the same asymmetry principle described in terrestrial plants applied to seagrasses. When a species pair is sharing a plot, the difference between their scores in PC1 (ΔFS _PC1_) showed that the higher the difference in size, the higher the probability that the bigger plant will exert space preemption. Applied to the actual species scores, the species with a higher score in PC1 did also have the highest probability of preemption. The traits correlated to PC1 can give fundamental insight on what the mechanisms behind the preemption were.

First, the only trait negatively correlated to PC1 was shoots/M. This indicates that traditionally classified ephemeral and pioneer species, namely *H. ovalis, H. uninervis, S. isoetifolium*, and *C. rotundata*, tend to show a lower probability of space preemption. This is clear when this result is compared to previous observations on competition or successional stages in seagrass meadows. In one of the first reports describing Mediterranean seagrass meadows, Aleem (1955) observed that when *P. oceanica* and *C. nodosa* shared the same space, the former would dominate the meadow. This idea was further developed by Young and Kirkman (1975), who described that seagrass meadows are in a state leading to bigger climax species. A similar work on succession is presented by Birch and Birch (1984), describing the development of a meadow from pioneer species (genus *Halophila*) to climax ones (in this case, *C. serrulata*). Secondly, four of the traits that were positively correlated to PC1 (leaf ML and MW, RhD, and root ML) are a proxy for the size of the plant, supporting the assumption that plant size tends to be the controlling factor in space preemption. Therefore, these traits could influence both light and inorganic nutrients preemption.

The case of light is relatively simple: seagrass tend to shade the understory of their canopy (Zieman et al., 1984), inhibiting the growth of plants that have higher light requirements than the light reaching them. As a directional resource, higher leaf ML and MW would proportionally block more light (Williams, 1987). Additionally, greater leaves/Sh indicate a denser canopy, and greater LMA indicates thicker leaves, further reducing the amount of light reaching the canopy understory. The case of inorganic nutrients is more complicated due to the non-directional nature of the resource. Intuitively, greater plant size translates into more tissue area for resource acquisition. Additionally, plants with longer root length would have access to deeper sediments and potentially new nutrient pools, inaccessible to smaller plants, which share the shallow sediment layer (Williams, 1990; Duarte et al., 1998). This establishes an extra advantage to bigger plants in addition to light preemption. This differentiation in belowground resource access also indicates niche differentiation, as it allows plants to access different resource pools, avoiding competition (Wilson, 1988; McConnaughay and Bazzaz, 1991; Duarte et al., 2000). However, the question of whether the selected traits indicate inorganic nutrient preemption remains unanswered, as the traits could indicate access to other nutrient pools, but not an interference of one seagrass species in the nutrient acquisition of a second seagrass species. Controlled experiments disentangling the confounding effects of light and inorganic nutrient preemption will prove fundamental to better understand which preemptable resource is more important for the final meadow configuration, or if the same preemptable resource is the driver of meadow configuration under changing environmental conditions. Alternatively, other traits related to seagrass physiology like growth rates and/or nutrient uptakes of both aboveground and belowground tissues could be better indicators of nutrient preemption in seagrasses.

The trophic states drove changes in the seagrass species traits. We showed how all species except *S. isoetifolium* decreased their size in the mesotrophic and eutrophic states in PC1. For a small species like *S. isoetifolium*, the increase in size relative to the other species translated in an increase in the probability of space preemption (Figure 9). Therefore, despite the same traits being responsible for competition, it is the increase in the size of *S. isoetifolium* that drove the increase in its capacity for space preemption. Similarly, this has been reported for the competition between *T. testudinum* and *H. wrightii* (Fourqurean et al., 1995). When the nutrient supply increased, equating the nutrient demands of *H. wrightii*, this species developed higher aboveground structures exerting light preemption on *T. testudinum*, resulting in the replacement of this species in the long term. It is therefore apparent that the traits driving competition for resources and, as a consequence, space preemption, remain the same even under inorganic nutrient excess. The change in the species that outcompetes another is a consequence of the species-specific trait response to nutrient excess. This highlights the importance of including environmental conditions together with trait metrics for the prediction of ecological processes (van der Plas et al., 2020).

It is however worth discussing which traits determine space preemption in radically different environments, e.g., subtidal and intertidal areas. Citing Aleem (1955) again, he described that, toward the intertidal area, *P. oceanica* is “at a minimum” and *C. nodosa* outcompetes the former. Zonation due to the tidal level is also mentioned in Phillips (1960). Harrison (1979) suggested that the intertidal area was dominated by *Zostera americana* due to this species being an r-strategist compared to the k-strategist *Z. marina*. Similarly, den Hartog (1970) reports that *Ruppia* sp. dominates in brackish waters, while other seagrass species preempts it from penetrating in waters with higher salinity. In the present study we found an effect of eutrophication on seagrass traits, but not in competition for space, likely because despite this effect, the traits determining preemption remained the same. However, other environmental factors can change which traits are important for species competition, i.e., traits that determine space preemption in subtidal seagrass meadows are not the same in intertidal meadows. As shown by Lan et al. (2005), *T. hemprichii* is able to dominate the intertidal area due to higher tolerance to air exposure than *H. uninervis*. This trait is unrelated to plant size or nutrient preemption. The selection of traits for the research question at hand and the relevant environmental drivers is therefore fundamental.

Despite the higher probability of space preemption for bigger seagrass plants, seagrass meadows in Unguja Island are generally mixed. This begs the question of how mixed meadows are maintained, despite the capacity of bigger species to preempt space from smaller species. Habitats are subjected to a disturbance regime that, potentially, can promote the seasonal or periodic growth of a species that generally would not exert space preemption (Turner, 1985). It is therefore apparent that seagrass meadows are able to maintain an array of species (Williams, 1990), in a successional series that is dynamic and does not end in the formation of a completely monospecific meadow. Additionally, other traits may be responsible for the persistence of a species despite its incapacity for space preemption. The study of more complex traits and physiological processes in seagrass could disentangle remaining questions in the competition for light and inorganic nutrients. In the case of competition for inorganic nutrients, the study of the nutrient demands and nutrient uptake rates (Angove et al., 2018) would give insight on seagrass competition under nutrient excess and limitation. In the case of the study of light preemption, the knowledge of the photosynthetic performance and pigment composition of seagrass plants would give insight into which species can withstand higher exposure to light or, inversely, shading.

Lastly, there are some relevant concepts that were not deeply discussed in this study that could open new and interesting lines of research in seagrass competition. First, plasticity and niche differentiation have been shown to be important drivers for the competition of terrestrial plants (Meilhac et al., 2020), showing how the trait differentiation in terrestrial grasslands influences competition. Due to the parallelism between terrestrial plant and seagrass competition presented in this study, it is expected that these phenomena will have an effect on competition among seagrass species as well. Second, competition with other primary producers was not included in this study, but there are a great number of reports showing that seagrass and benthic macroalgae compete for resources and space (Dethier, 1984; Turner, 1985; Davis and Fourqurean, 2001; Taplin et al., 2005; Moreira-Saporiti et al., 2021). While traits important for interspecific competition with benthic algae may be the same as for competition with seagrass, this may not be the case. This question is of great importance for the understanding of invasive algae colonization in seagrass meadows (De Villèle and Verlaque, 1995; Ceccherelli and Cinelli, 1997).

## Conclusions

This study advances our understanding of the ecological processes that shape the configuration of seagrass meadows by describing competition using a trait-based approach. The traits linked to light and inorganic nutrients preemption that were used here (leaf length and width, rhizome diameter, shoots meter^−1^, among others) define the functional strategy of seagrass species by showing a trade-off between size and density of shoots. We found that the probability of space preemption was positively correlated with the traits' indicative of larger plant size. This indicates that competitive interactions in subtidal seagrass are asymmetrical and favor larger seagrass species, which exert a negative effect on smaller species without a reciprocal negative response.

## Data Availability Statement

All the data used in this article is openly accessible in the PANGAEA database under a Creative Commons license (CC-BY) via the links below:

Saporiti, Agustín Moreira; Teichberg, Mirta (2021): Chlorophyll a in the water column in Unguja Island (Zanzibar Archipealgo, Tanzania) sampled in 2016. PANGAEA, https://doi.org/10.1594/PANGAEA.933460

Saporiti, Agustín Moreira; Teichberg, Mirta (2021): Macroalgae biomass in Unguja Island (Zanzibar Archipealgo, Tanzania) sampled in 2016. PANGAEA, https://doi.org/10.1594/PANGAEA.932885

Saporiti, Agustín Moreira; Teichberg, Mirta (2021): Dissolved inorganic nitrogen and phosphate in pore water in Unguja Island (Zanzibar Archipealgo, Tanzania) sampled in 2016. PANGAEA, https://doi.org/10.1594/PANGAEA.932894

Saporiti, Agustín Moreira; Teichberg, Mirta (2021): ∂15N in the sediment in Unguja Island (Zanzibar Archipealgo, Tanzania) sampled in 2016. PANGAEA, https://doi.org/10.1594/PANGAEA.932895

Saporiti, Agustín Moreira; Teichberg, Mirta (2021): Seagrass abundance in Unguja Island (Zanzibar Archipealgo, Tanzania) sampled in 2016. PANGAEA, https://doi.org/10.1594/PANGAEA.932896

Saporiti, Agustín Moreira; Teichberg, Mirta (2021): Seagrass traits (ramet level) in Unguja Island (Zanzibar Archipealgo, Tanzania) sampled in 2016. PANGAEA, https://doi.org/10.1594/PANGAEA.932898

Saporiti, Agustín Moreira; Teichberg, Mirta (2021): Seagrass traits (shoot level) in Unguja Island (Zanzibar Archipealgo, Tanzania) sampled in 2016. PANGAEA, https://doi.org/10.1594/PANGAEA.932899

Saporiti, Agustín Moreira; Teichberg, Mirta (2021): Leaf mass area in Unguja Island (Zanzibar Archipealgo, Tanzania) sampled in 2016. PANGAEA, https://doi.org/10.1594/PANGAEA.932901

## Author Contributions

MT arranged the project funding. AM-S, MT, and IV designed the sampling campaign to obtain the data for this manuscript and carried out the sampling campaign. AM-S and IV processed and analyzed the samples. AM-S carried out the data analysis with guidance from SB and wrote the first version of the manuscript. MT, IV, SB, EB, and MM made significant contributions to the manuscript and critically revised the different versions of the manuscript. All authors contributed to the article and approved the submitted version.

## Funding

IV was awarded with a postdoctoral contract of Xunta de Galicia (Consellería de Educación, Universidad e Formación Profesional) postdoctoral program (ED481B-2016/189-0) and Juan de la Cierva-Incorporación postdoctoral program (IJC2019-040554-I). This study was part of the project Seagrass and Macroalgal Community Dynamics and Performance under Environmental Change (SEAMAC) (Deutsche Forschungsgemeinschaft, DFG, TE 1046/3-1) awarded to MT.

## Conflict of Interest

The authors declare that the research was conducted in the absence of any commercial or financial relationships that could be construed as a potential conflict of interest.

## Publisher's Note

All claims expressed in this article are solely those of the authors and do not necessarily represent those of their affiliated organizations, or those of the publisher, the editors and the reviewers. Any product that may be evaluated in this article, or claim that may be made by its manufacturer, is not guaranteed or endorsed by the publisher.
