# Supplementary material for: Local Victory: Assessing Interspecific Competition in Seagrass From a Trait-Based Perspective
Source: Front Plant Sci. 2021 Nov 2;12:709257. doi: 10.3389/fpls.2021.709257 (PMC8593471; doi:10.3389/fpls.2021.709257)
Supplement: Supplementary file 1 [file Data_Sheet_1.docx]

**Supplementary material 1. Raw trait data**

All data is presented as Mean ± Standard Error

| Species | Sites | Sample size (n) | Belowground structure traits | | | Canopy forming traits | | | | | |
| --- | --- | --- | --- | --- | --- | --- | --- | --- | --- | --- | --- |
|  |  |  | RhD (cm) | Roots/M | Root ML (cm) | Shoots/M | VR length (cm) | Leaves/Sh | Leaf ML (cm) | Leaf MW (cm) | LMA (g DW/cm^2^) |
| CR | Marumbi | 2 | 0.28±0.03 | 76.58±0.34 | 11.45±2.45 | 26.73±0.63 | 3.90±1.7 | 2.00±0.00 | 13.05±0.45 | 0.45±0.00 | 3.30±0.25 |
|  | Chapwani | 10 | 0.25±0.01 | 244.63±64.07 | 8.14±0.51 | 55.76±4.28 | 1.59±0.24 | 2.94±0.14 | 6.30±0.45 | 0.27±0.01 | 3.39±0.15 |
|  | Bweleo | 20 | 0.28±0.01 | 88.30±9.96 | 10.34±1.01 | 29.54±2.18 | 0.83±0.32 | 2.62±0.10 | 8.93±0.51 | 0.39±0.01 | 3.16±0.09 |
|  | Harbor | 19 | 0.27±0.01 | 122.06±18.61 | 10.57±0.78 | 37.79±2.08 | 1.87±0.25 | 2.75±0.12 | 10.63±0.66 | 0.35±0.01 | 3.80±0.26 |
| CS | Changuu | 15 | 0.34±0.02 | 107.61±26.13 | 14.21±0.96 | 37.10±3.69 | 0.57±0.12 | 3.09±0.05 | 7.82±0.59 | 0.87±0.02 | 2.97±0.15 |
|  | Chapwani | 20 | 0.35±0.02 | 245.27±56.38 | 12.20±1.09 | 32.47±2.72 | 2.28±0.36 | 3.23±0.08 | 8.84±0.50 | 0.90±0.02 | 3.05±0.09 |
|  | Bweleo | 19 | 0.34±0.01 | 174.59±33.51 | 10.51±0.80 | 35.91±5.12 | 1.17±0.33 | 3.08±0.11 | 10.18±0.70 | 0.93±0.03 | 2.70±0.08 |
|  | Fumba | 20 | 0.38±0.01 | 504.18±107.43 | 11.40±0.85 | 49.90±5.92 | 1.32±0.39 | 3.18±0.08 | 10.38±0.66 | 0.96±0.02 | 2.52±0.06 |
| EA | Marumbi | 19 | 1.09±0.04 | 236.37±4.66 | 10.96±1.02 | 37.81±0.21 | 0.00±0.00 | 3.77±0.12 | 44.48±2.47 | 1.57±0.06 | 5.21±0.09 |
|  | Fumba | 20 | 1.00±0.03 | 236.37±0.00 | 9.57±0.76 | 37.81±0.00 | 0.00±0.00 | 3.30±0.14 | 43.44±2.32 | 1.32±0.04 | 4.33±0.12 |
| HO | Chapwani | 13 | 0.11±0.01 | 50.39±4.63 | 3.14±0.37 | 54.81±3.92 | 0.79±0.09 | 1.72±0.08 | 1.08±0.04 | 0.50±0.03 | 1.67±0.12 |
|  | Bweleo | 20 | 0.12±0.01 | 50.21±4.54 | 3.39±0.29 | 51.41±5.36 | 1.34±0.10 | 1.93±0.04 | 1.78±0.11 | 0.78±0.03 | 1.54±0.07 |
| HU | Mangroves | 10 | 0.17±0.01 | 230.79±26.08 | 8.78±0.35 | 42.49±2.57 | 0.50±0.09 | 2.91±0.16 | 5.26±0.57 | 0.23±0.03 | 3.68±0.16 |
|  | Marumbi | 15 | 0.18±0.01 | 439.94±52.64 | 7.19±0.32 | 50.01±2.79 | 3.05±0.49 | 2.62±0.13 | 10.97±0.71 | 0.25±0.01 | 3.92±0.24 |
|  | Chapwani | 20 | 0.15±0.01 | 616.92±75.54 | 6.29±0.57 | 58.68±4.62 | 0.71±0.21 | 2.46±0.13 | 6.95±0.58 | 0.20±0.01 | 3.43±0.17 |
|  | Bweleo | 20 | 0.16±0.01 | 357.01±59.31 | 6.10±0.62 | 43.31±2.76 | 1.12±0.26 | 2.77±0.17 | 7.41±0.35 | 0.22±0.01 | 2.54±0.08 |
| SI | Changuu | 20 | 0.20±0.01 | 217.73±20.92 | 7.23±0.37 | 53.98±2.94 | 0.43±0.04 | 1.36±0.07 | 6.23±0.44 | 0.13±0.00 | 2.36±0.07 |
|  | Chapwani | 10 | 0.22±0.01 | 298.94±45.43 | 7.23±0.41 | 59.27±7.05 | 1.36±0.46 | 1.52±0.08 | 6.84±0.59 | 0.14±0.01 | 2.96±0.19 |
|  | Fumba | 20 | 0.25±0.01 | 246.78±22.74 | 8.13±0.42 | 63.07±3.61 | 0.88±0.13 | 1.82±0.09 | 10.15±0.78 | 0.17±0.01 | 2.67±0.10 |
| TC | Changuu | 20 | 0.50±0.02 | 140.30±10.55 | 14.30±0.80 | 37.55±2.20 | 5.54±0.59 | 6.41±0.20 | 7.47±0.36 | 1.02±0.04 | 3.00±0.27 |
| TH | Changuu | 20 | 0.30±0.01 | 84.03±9.76 | 7.75±0.39 | 19.06±1.10 | 0.67±0.15 | 3.85±0.19 | 7.15±0.49 | 0.57±0.03 | 4.39±0.33 |
|  | Mangroves | 20 | 0.36±0.02 | 317.85±27.38 | 5.45±0.40 | 29.53±2.48 | 1.56±0.26 | 4.98±0.27 | 6.10±0.45 | 0.71±0.04 | 5.05±0.50 |
|  | Marumbi | 20 | 0.41±0.01 | 194.89±39.07 | 9.72±0.75 | 17.38±1.42 | 1.92±0.33 | 3.83±0.14 | 13.20±0.89 | 0.87±0.05 | 4.89±0.11 |
|  | Chapwani | 19 | 0.31±0.02 | 68.01±9.97 | 8.43±0.82 | 17.57±1.23 | 0.91±0.16 | 4.01±0.19 | 5.89±0.47 | 0.54±0.02 | 4.21±0.13 |
|  | Bweleo | 20 | 0.36±0.02 | 127.01±16.50 | 7.82±0.58 | 13.88±0.96 | 2.71±0.48 | 3.49±0.14 | 11.82±0.57 | 0.80±0.02 | 3.72±0.10 |
|  | Fumba | 20 | 0.31±0.01 | 160.88±46.55 | 8.01±0.64 | 16.88±1.65 | 2.08±0.34 | 4.13±0.24 | 9.92±0.71 | 0.77±0.03 | 3.65±0.15 |
|  | Harbor | 20 | 0.35±0.01 | 138.06±29.23 | 6.32±0.46 | 19.53±1.82 | 2.16±0.28 | 3.58±0.15 | 16.36±0.64 | 0.76±0.02 | 4.25±0.26 |

**Supplementary material 2. Site clustering in trophic states**

Silhouette width of the sites in the trophic clusters

**
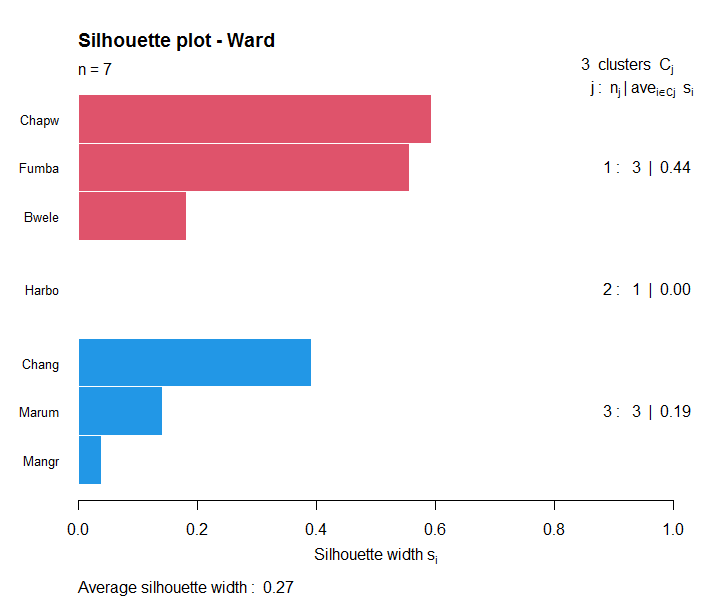
**

**Supplementary material 3. Ordination diagnostics**

**1. Quality of representation of the PCA**

**1.1. mSD calculation:**

**
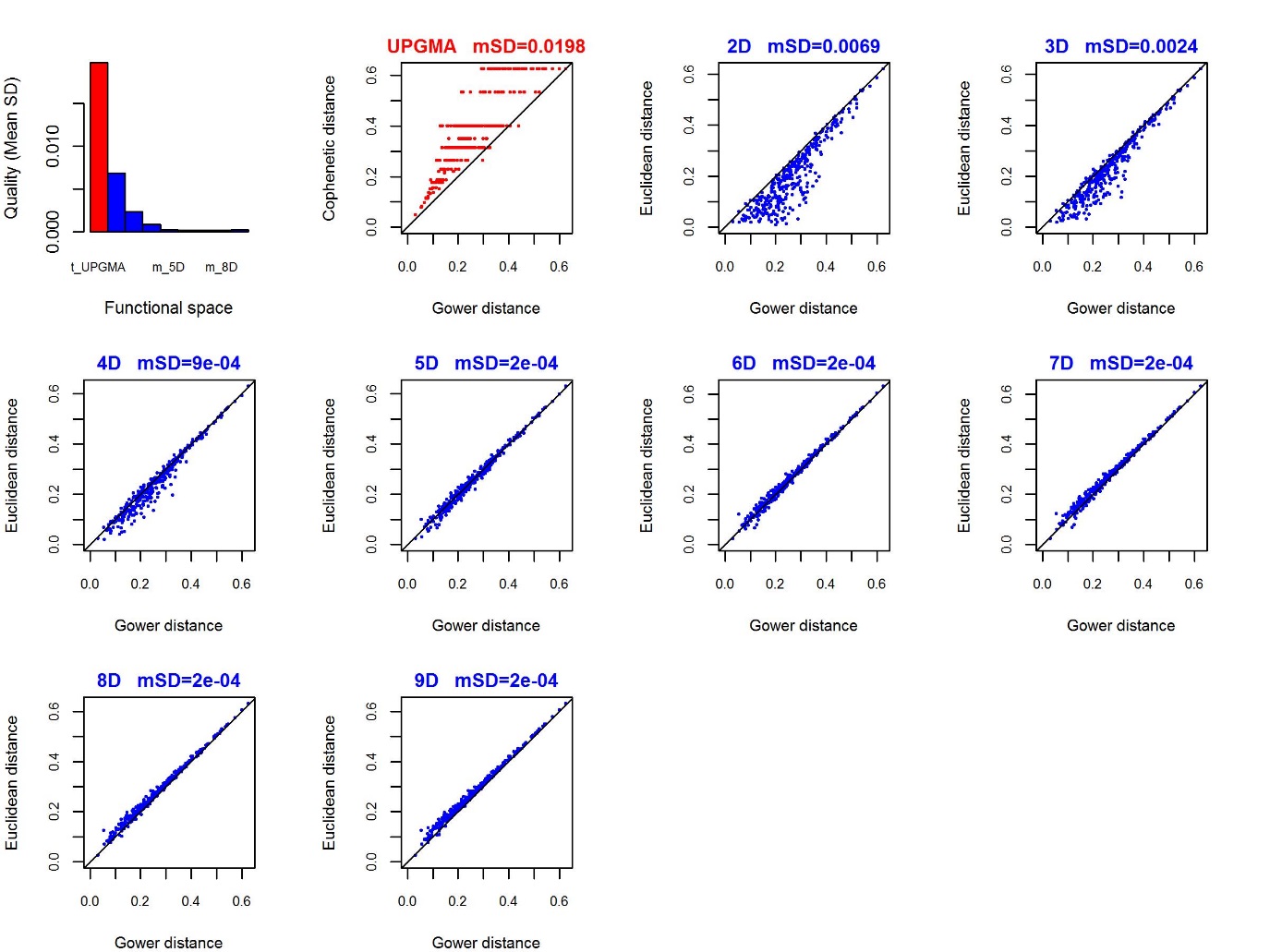
**

**1.2. Comparison of mSDs for principal components retention:**


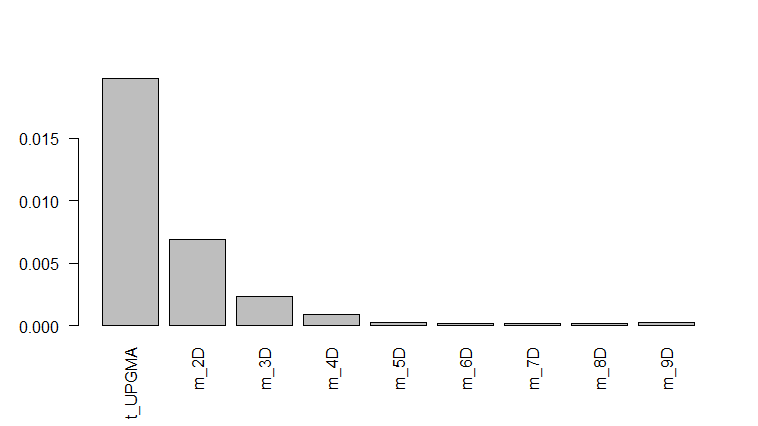


**Supplementary material 4. Effect of difference in the functional strategy of seagrass on space preemption**

**1. Species pairs: Number of plots per species pair and site**

| Trophic state | Site | Species pair | | Number of plots | Preemption | No preemption |
| --- | --- | --- | --- | --- | --- | --- |
| Oligotrophic | Changuu | *C. serrulata* | *S. isoetifolium* | 16 | 12 | 4 |
|  |  |  | *T. ciliatum* | 10 | 4 | 6 |
|  |  |  | *T. hemprichii* | 17 | 6 | 11 |
|  |  | *S. isoetifolium* | *C. serrulata* | 16 | 3 | 13 |
|  |  |  | *T. ciliatum* | 11 | 2 | 9 |
|  |  |  | *T. hemprichii* | 23 | 5 | 18 |
|  |  | *T. ciliatum* | *C. serrulata* | 10 | 5 | 5 |
|  |  |  | *S. isoetifolium* | 11 | 9 | 2 |
|  |  |  | *T. hemprichii* | 14 | 8 | 6 |
|  |  | *T. hemprichii* | *C. serrulata* | 17 | 7 | 10 |
|  |  |  | *S. isoetifolium* | 23 | 14 | 9 |
|  |  |  | *T. ciliatum* | 14 | 4 | 10 |
|  | Marumbi | *C. rotundata* | *T. hemprichii* | 8 | 1 | 7 |
|  |  | *E. acoroides* | *T. hemprichii* | 10 | 6 | 4 |
|  |  | *H. uninervis* | *T. hemprichii* | 13 | 4 | 9 |
|  |  | *T. hemprichii* | *C. rotundata* | 8 | 6 | 2 |
|  |  |  | *E. acoroides* | 10 | 2 | 8 |
|  |  |  | *H. uninervis* | 13 | 7 | 6 |
| Mesotrophic | Bweleo | *C. rotundata* | *C. serrulata* | 6 | 0 | 6 |
|  |  |  | *H. ovalis* | 10 | 3 | 7 |
|  |  |  | *H. uninervis* | 10 | 2 | 8 |
|  |  |  | *T. hemprichii* | 9 | 1 | 8 |
|  |  | *C. serrulata* | *C. rotundata* | 6 | 4 | 2 |
|  |  |  | *H. uninervis* | 17 | 11 | 6 |
|  |  |  | *T. hemprichii* | 17 | 10 | 7 |
|  |  | *H. ovalis* | *C. rotundata* | 10 | 1 | 9 |
|  |  |  | *H. uninervis* | 13 | 7 | 6 |
|  |  |  | *T. hemprichii* | 7 | 1 | 6 |
|  |  | *H. uninervis* | *C. rotundata* | 10 | 6 | 4 |
|  |  |  | *C. serrulata* | 17 | 4 | 13 |
|  |  |  | *H. ovalis* | 13 | 4 | 9 |
|  |  |  | *T. hemprichii* | 18 | 4 | 14 |
|  |  | *T. hemprichii* | *C. rotundata* | 9 | 5 | 4 |
|  |  |  | *C. serrulata* | 187 | 4 | 13 |
|  |  |  | *H. ovalis* | 7 | 5 | 2 |
|  |  |  | *H. uninervis* | 18 | 10 | 8 |
|  | Chapwani | *C. rotundata* | *H. uninervis* | 5 | 3 | 2 |
|  |  |  | *T. hemprichii* | 6 | 1 | 5 |
|  |  | *C. serrulata* | *S. isoetifolium* | 8 | 5 | 3 |
|  |  |  | *T. hemprichii* | 10 | 5 | 5 |
|  |  | *H. ovalis* | *H. uninervis* | 6 | 0 | 6 |
|  |  |  | *T. hemprichii* | 5 | 0 | 5 |
|  |  | *H. uninervis* | *C. rotundata* | 5 | 1 | 4 |
|  |  |  | *H. ovalis* | 6 | 6 | 0 |
|  |  |  | *S. isoetifolium* | 5 | 1 | 4 |
|  |  |  | *T. hemprichii* | 19 | 1 | 18 |
|  |  | *S. isoetifolium* | *C. serrulata* | 8 | 2 | 6 |
|  |  |  | *H. uninervis* | 5 | 4 | 1 |
|  |  |  | *T. hemprichii* | 12 | 3 | 9 |
|  |  | *T. hemprichii* | *C. rotundata* | 6 | 5 | 1 |
|  |  |  | *C. serrulata* | 10 | 5 | 5 |
|  |  |  | *H. ovalis* | 5 | 5 | 0 |
|  |  |  | *H. uninervis* | 19 | 18 | 1 |
|  |  |  | *S. isoetifolium* | 12 | 9 | 3 |
|  | Fumba | *E. acoroides* | *S. isoetifolium* | 5 | 2 | 3 |
|  |  |  | *T. hemprichii* | 6 | 4 | 2 |
|  |  | *S. isoetifolium* | *E. acoroides* | 5 | 3 | 2 |
|  |  | *T. hemprichii* | *E. acoroides* | 6 | 2 | 4 |
| Eutrophic | Harbor | *C. rotundata* | *T. hemprichii* | 5 | 2 | 3 |
|  |  | *T. hemprichii* | *C. rotundata* | 5 | 3 | 2 |

**Supplementary material 5. Seagrass cover and pairwise preemption: pairwise comparisons**

**1. Model 1: Pairwise differences in the % cover of seagrass species within trophic state**

| Response variable | Trophic state | Species pairs | z-value | p-value |
| --- | --- | --- | --- | --- |
| % Seagrass  cover | Oligotrophic | **CR-CS** | **2.884** | **0.0039**** |
|  |  | **CR-EA** | **2.092** | **0.0364*** |
|  |  | CR-HU | 1.134 | 0.2570 |
|  |  | CR-SI | 0.483 | 0.6289 |
|  |  | **CR-TC** | **2.783** | **0.0053**** |
|  |  | **CR-TH** | **2.519** | **0.0117*** |
|  |  | CS-EA | 0.567 | 0.5706 |
|  |  | **CS-HU** | **2.063** | **0.0391*** |
|  |  | **CS-SI** | **3.485** | **0.0004***** |
|  |  | CS-TC | 0.085 | 0.9324 |
|  |  | CS-TH | 1.004 | 0.3152 |
|  |  | EA-HU | 1.198 | 0.2307 |
|  |  | **EA-SI** | **2.175** | **0.0297*** |
|  |  | EA-TC | 0.489 | 0.6249 |
|  |  | EA-TH | 0.108 | 0.9138 |
|  |  | HU-SI | 0.942 | 0.3461 |
|  |  | HU-TC | 1.945 | 0.0518 |
|  |  | HU-TH | 1.548 | 0.1216 |
|  |  | **SI-TC** | **3.301** | **0.0009***** |
|  |  | **SI-TH** | **3.297** | **0.0009***** |
|  |  | TC-TH | 0.866 | 0.3867 |
|  | Mesotrophic | **CR-CS** | **4.931** | **0.0000***** |
|  |  | **CR-EA** | **2.619** | **0.0088**** |
|  |  | CR-HO | 0.548 | 0.5834 |
|  |  | CR-HU | 0.471 | 0.6375 |
|  |  | **CR-SI** | **2.717** | **0.0065**** |
|  |  | **CR-TH** | **4.054** | **0.0000***** |
|  |  | CS-EA | 0.063 | 0.9490 |
|  |  | **CS-HO** | **5.620** | **0.0000***** |
|  |  | **CS-HU** | **5.602** | **0.0000***** |
|  |  | CS-SI | 1.513 | 0.1300 |
|  |  | CS-TH | 1.510 | 0.1310 |
|  |  | **EA-HO** | **2.933** | **0.0033**** |
|  |  | **EA-HU** | **2.467** | **0.0136*** |
|  |  | EA-SI | 0.932 | 0.3512 |
|  |  | EA-TH | 0.712 | 0.4763 |
|  |  | HO-HU | 1.129 | 0.2588 |
|  |  | **HO-SI** | **3.264** | **0.0011**** |
|  |  | **HO-TH** | **4.790** | **0.0000***** |
|  |  | **HU-SI** | **2.737** | **0.0062**** |
|  |  | **HU-TH** | **4.690** | **0.0000***** |
|  |  | SI-TH | 0.498 | 0.6183 |

**2. Model 2: Pairwise differences in the % cover within a seagrass species among trophic states**

| Response  variable | Species | Trophic states | z-value | p-value |
| --- | --- | --- | --- | --- |
| %Seagrass  cover | *Cymodocea rotundata* | Oligotrophic-Mesotrophic | 0.243 | 0.8082 |
|  |  | **Oligotrophic-Eutrophic** | **2.437** | **0.0148*** |
|  |  | **Mesotrophic-Eutrophic** | **3.044** | **0.0023**** |
|  | *Halodule uninervis* | **Oligotrophic-Mesotrophic** | **1.982** | **0.0475*** |

**3. Model 3: Pairwise differences in the probability of preemption of species within trophic state**

| Response variable | Trophic state | Species pairs | z-value | p-value |
| --- | --- | --- | --- | --- |
| Probability of preemption | Oligotrophic | CR-CS | 1.384 | 0.167 |
|  |  | CR-EA | 1.102 | 0.270 |
|  |  | CR-HU | 0.745 | 0.456 |
|  |  | CR-SI | 0.531 | 0.596 |
|  |  | **CR-TC** | **1.978** | **0.048*** |
|  |  | CR-TH | 1.219 | 0.223 |
|  |  | CS-EA | 0.071 | 0.943 |
|  |  | CS-HU | 0.696 | 0.486 |
|  |  | **CS-SI** | **3.068** | **0.002**** |
|  |  | CS-TC | 1.033 | 0.301 |
|  |  | CS-TH | 0.433 | 0.664 |
|  |  | EA-HU | 0.470 | 0.638 |
|  |  | **EA-SI** | **2.048** | **0.040*** |
|  |  | EA-TC | 0.779 | 0.435 |
|  |  | EA-TH | 0.187 | 0.851 |
|  |  | HU-SI | 1.698 | 0.089 |
|  |  | HU-TC | 1.461 | 0.144 |
|  |  | HU-TH | 0.449 | 0.653 |
|  |  | **SI-TC** | **3.845** | **0.000***** |
|  |  | **SI-TH** | **3.073** | **0.002**** |
|  |  | TC-TH | 1.562 | 0.118 |
|  | Mesotrophic | **CR-CS** | **4.000** | **0.000***** |
|  |  | CR-EA | 1.732 | 0.083 |
|  |  | CR-HO | 0.320 | 0.749 |
|  |  | CR-HU | 0.534 | 0.593 |
|  |  | **CR-SI** | **2.215** | **0.026*** |
|  |  | **CR-TH** | **4.038** | **0.000***** |
|  |  | CS-EA | 0.800 | 0.423 |
|  |  | **CS-HO** | **4.170** | **0.000***** |
|  |  | **CS-HU** | **4.283** | **0.000***** |
|  |  | CS-SI | 1.476 | 0.139 |
|  |  | CS-TH | 0.358 | 0.720 |
|  |  | EA-HO | 1.928 | 0.053 |
|  |  | EA-HU | 1.512 | 0.130 |
|  |  | EA-SI | 0.167 | 0.867 |
|  |  | EA-TH | 0.643 | 0.520 |
|  |  | HO-HU | 0.876 | 0.381 |
|  |  | **HO-SI** | **2.451** | **0.014*** |
|  |  | **HO-TH** | **4.203** | **0.000***** |
|  |  | **HU-SI** | **2.049** | **0.040*** |
|  |  | **HU-TH** | **4.481** | **0.000***** |
|  |  | SI-TH | 1.306 | 0.191 |

**4. Model 4: Pairwise differences in the probability of preemption within a seagrass species among trophic states**

| Response  variable | Species | Trophic states | z-value | p-value |
| --- | --- | --- | --- | --- |
| Probability of preemption | *Syringodium isoetifolium* | **Oligotrophic-Mesotrophic** | **2.622** | **0.0087**** |

**Supplementary material 6. Results of PCA**

**1. Eigenvalues of the PCs of the multidimensional space:**

| **Principal component** | **Eigenvalue** |
| --- | --- |
| PC 1 | 3.81 |
| PC 2 | 1.82 |
| PC 3 | 1.11 |
| PC 4 | 1.01 |
| PC 5 | 0.50 |
| PC 6 | 0.41 |

**2. Functional traits: Test of significant correlations to the six PCs of the multidimensional space**

Following Yamamoto et al. (2014), the t-statistic of the correlation can be calculated as:

$$t=\frac{{FT}_{corr}*\sqrt{n-2}}{\sqrt{1-{({FT}_{corr})}^{2}}}$$

Where FT_corr_ is the correlation value of the functional traits to the multidimensional space d and n is the number of replicates. The t-values for each functional trait and PC can be found in the following table. Significance was set at p<0.05 and is marked in boldface and underlined.

| Functional trait | t – value (df = 26, t-critic = 2.056) | | | | | |
| --- | --- | --- | --- | --- | --- | --- |
|  | PC 1 | PC 2 | PC 3 | PC 4 | PC 5 | PC 6 |
| Rhizome diameter | **9.389** | **2.361** | 0.174 | -0.744 | 0.287 | 0.446 |
| Root maximum length | **3.468** | -1.001 | **2.656** | **-2.372** | **-2.649** | -1.072 |
| Shoots/Meter | **-4.121** | **3.177** | **2.293** | -1.059 | 0.689 | 0.249 |
| Roots/Meter | -0.914 | **2.847** | **4.541** | **2.806** | 0.161 | -0.536 |
| Leaves/Shoot | **5.181** | **-2.458** | 1.011 | 1.509 | 1.406 | -1.306 |
| Vertical rhizome length | 0.703 | **-5.621** | **2.830** | -0.123 | 0.537 | **2.421** |
| Leaf maximum length | **5.709** | **3.636** | -0.110 | -0.490 | -0.224 | 1.518 |
| Leaf maximum width | **8.093** | 0.526 | -0.033 | -1.500 | 1.952 | -0.666 |
| Leaf mass area | **5.095** | 0.348 | -0.714 | **3.944** | -1.294 | 0.482 |

**3. Species centroids across the study area in the rest of the PCs:**

PC4 (Tables 4 and 5) indicated that only *H. uninervis* and *T. hemprichii* showed a certain correlation between root density and length, which in previous PCs were inversely correlated. In the last two PCs (PC 5 and 6), there were only unique trait correlations and the portion of explained unconstrained variability was very low (5.62% and 4.62% respectively, Table 5). For this reason, the species centroids showed no clear trends and their interpretation was, therefore, difficult.

**4. Ordination results in the rest of the PCs**

Results of the ordination of traits. On the left, the coordinates (scores) of the seagrass species per site in the multidimensional space (see Table 4 for species acronyms) and on the right the correlations of each functional trait with the dimensions of the ordination (Table 5 for trait nomenclature and PCA output). Colors represent the trophic state.

| 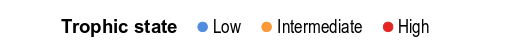 | |
| --- | --- |
| a) | |
| 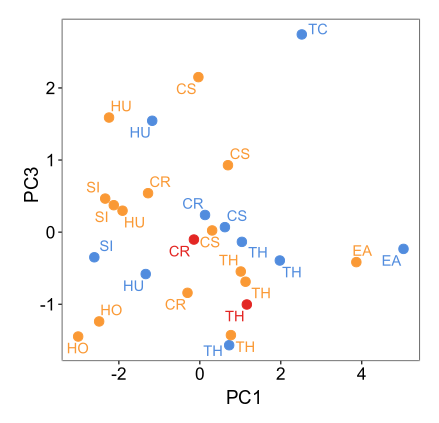 | 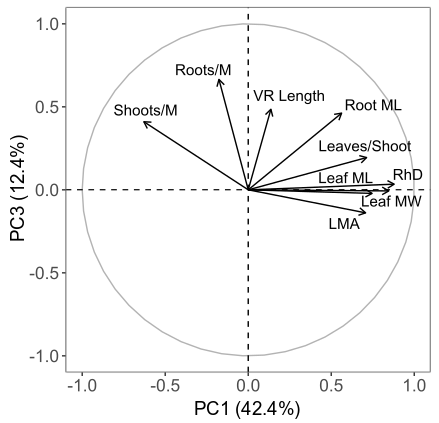 |
| b) | |
| 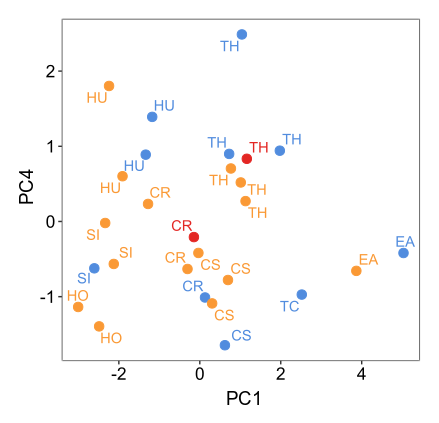 | 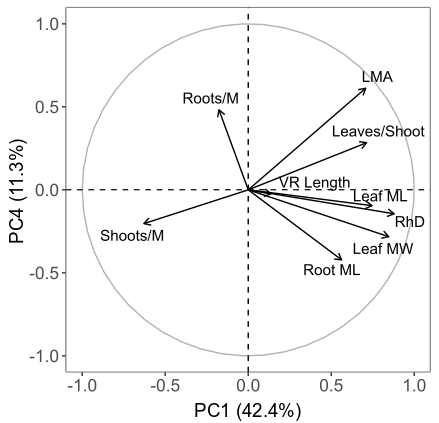 |

| 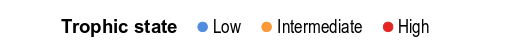 | |
| --- | --- |
| c) | |
| 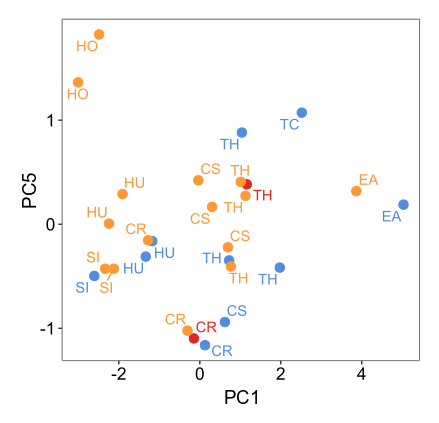 | 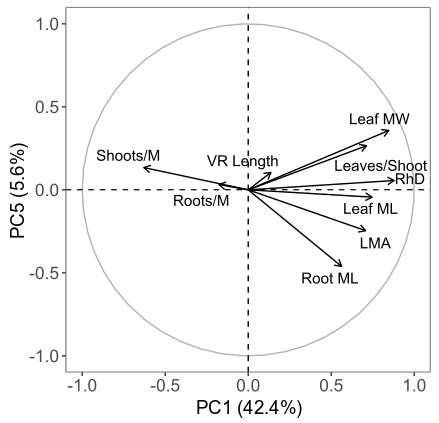 |
| d) | |
| 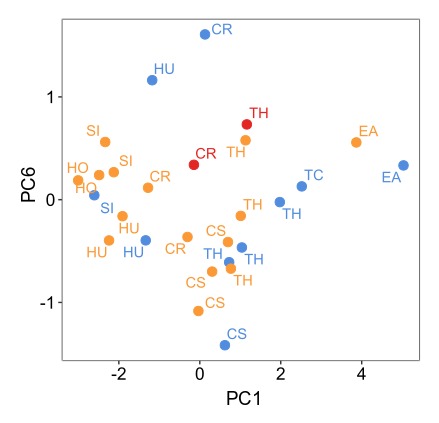 | 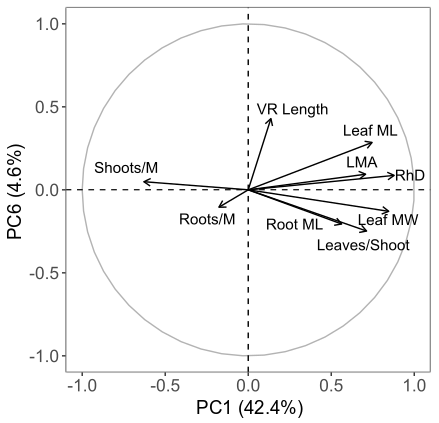 |
| e) | |
| 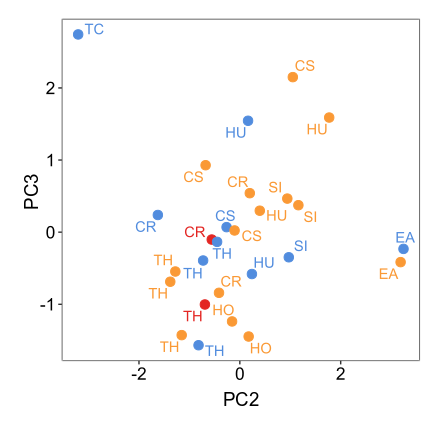 | 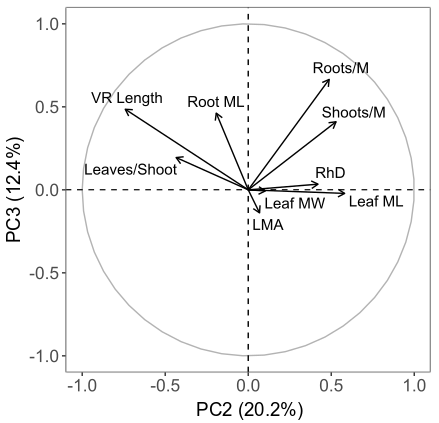 |

| 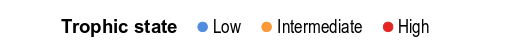 | |
| --- | --- |
| f) | |
| 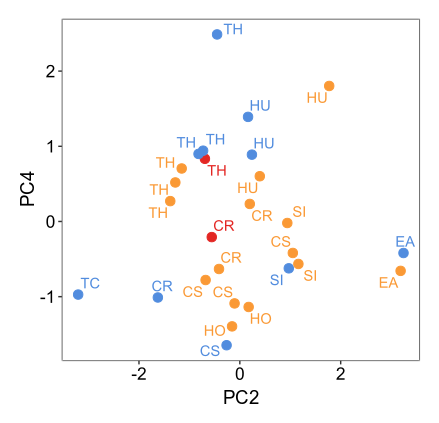 | 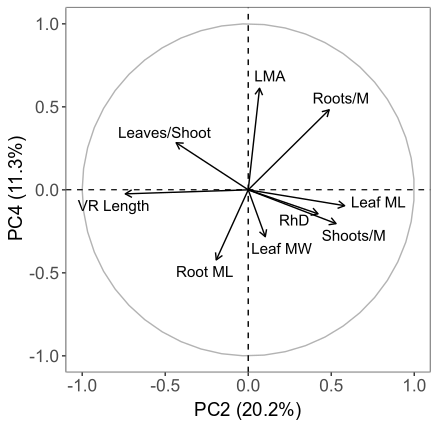 |
| g) | |
| 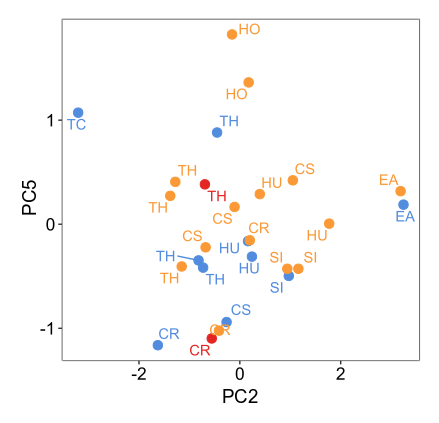 | 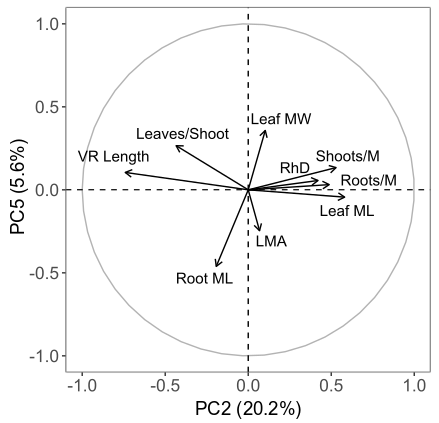 |
| h) | |
| 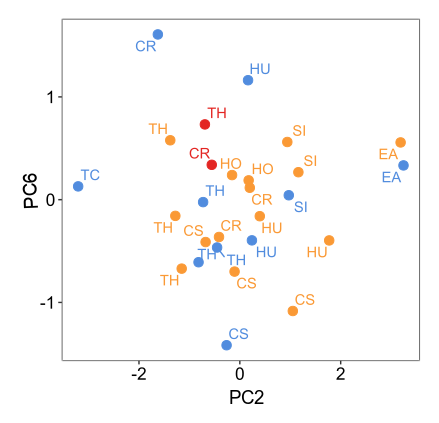 | 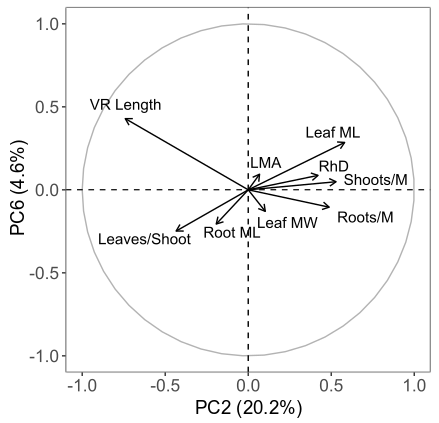 |
| 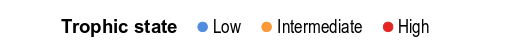 | |
| i) | |
| 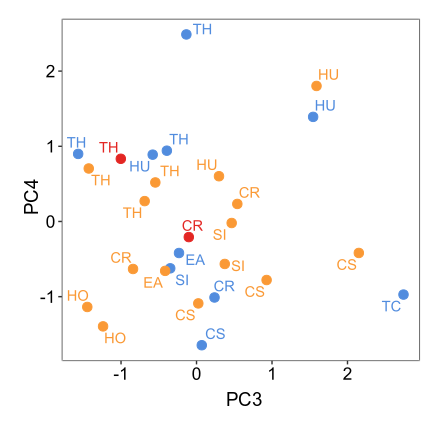 | 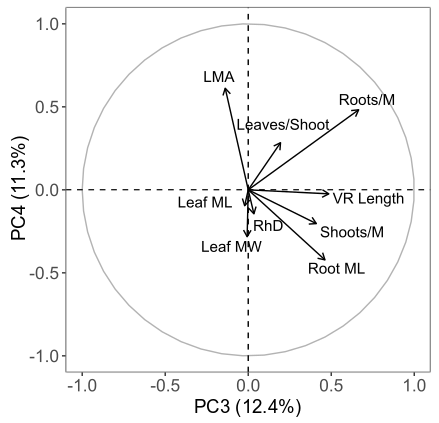 |
| j) | |
| 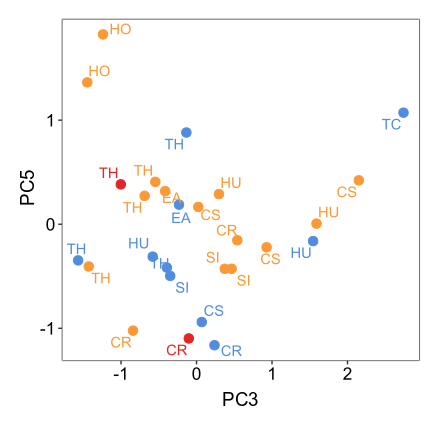 | 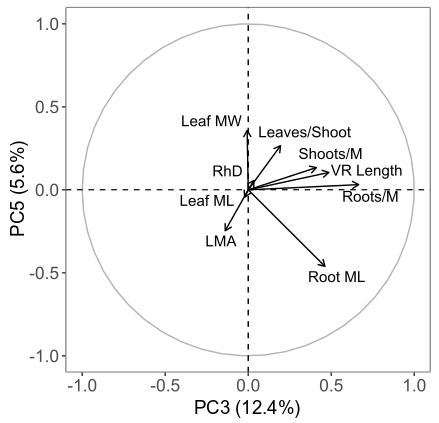 |
| k) | |
| 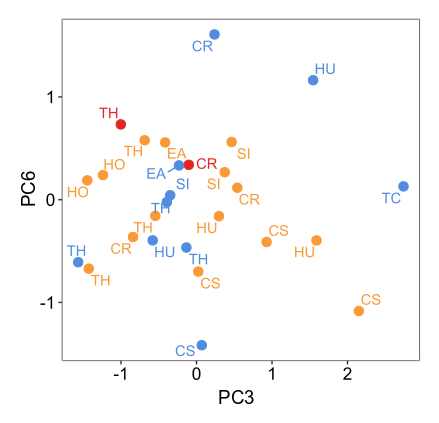 | 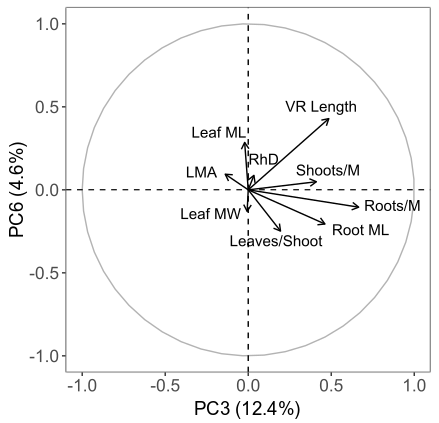 |

| 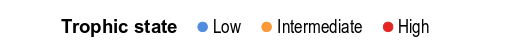 | |
| --- | --- |
| l) | |
| 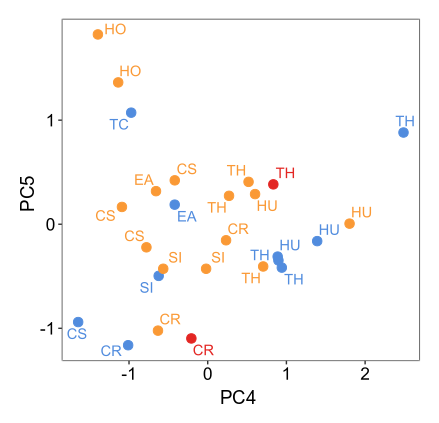 | 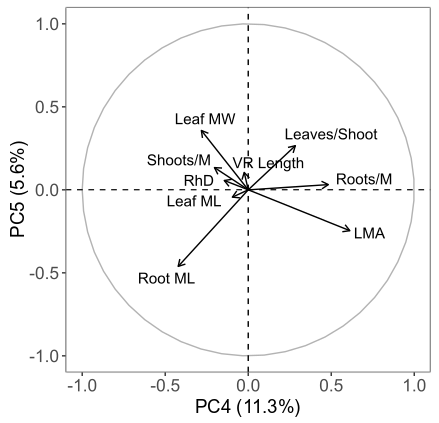 |
| m) | |
| 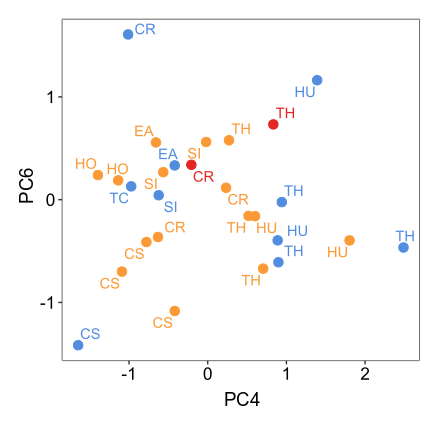 | 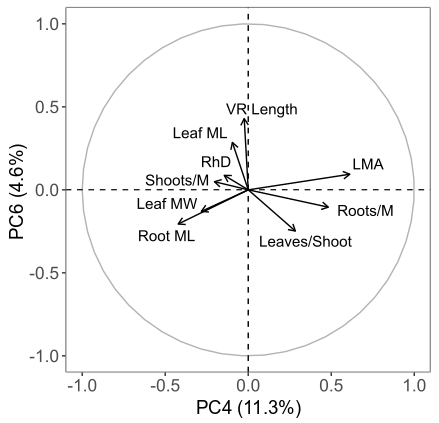 |
| n) | |
| 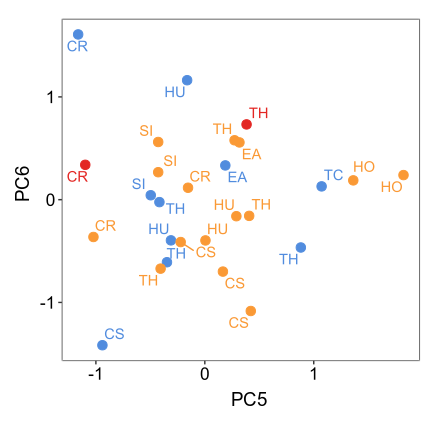 | 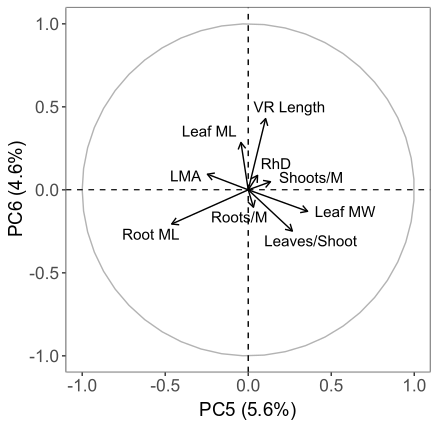 |

**Supplementary material 7. Trophic state effect on species centroids in all PCs**

Orange bars represent the difference between the species centroids in the oligotrophic and mesotrophic states, and the red bars the difference between the oligotrophic and eutrophic states.

**
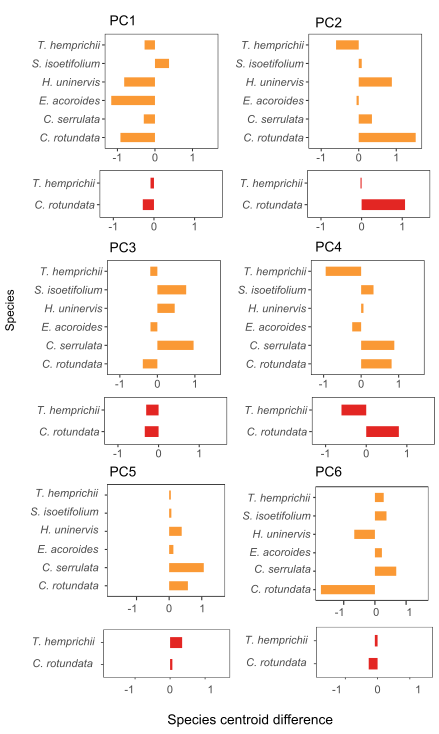
**

**Supplementary material 8. Probability of space preemption in PC2, PC3, PC4 and PC6**

**Results of effect of ΔFS of PC2, PC3, PC4 and PC6**

**
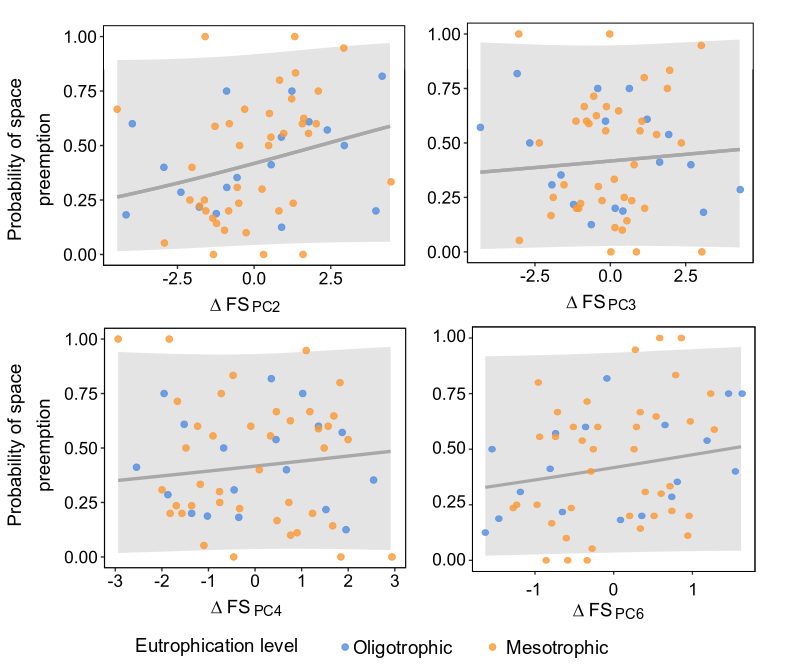
**
